# Exploring the dynamic adaptive responses of *Epimedium pubescens* to phosphorus deficiency by Integrated transcriptome and miRNA analysis

**DOI:** 10.1186/s12870-024-05063-y

**Published:** 2024-05-30

**Authors:** Shangnian Liu, Xiaojing An, Chaoqun Xu, Baolin Guo, Xianen Li, Caixia Chen, Dongmei He, De Xu, Yi Li

**Affiliations:** 1 School of Pharmacy, State Key Laboratory of Characteristic Chinese Medicine Resources in Southwest China, Chengdu University of Traditional Chinese Medicine, Chengdu, 611137 China; 2 Key Laboratory of Bioactive Substances and Resources Utilization of Chinese Herbal Medicines, Ministry of Education & National Engineering Laboratory for Breeding of Endangered Medicinal Materials, Institute of Medicinal Plant Development, Peking Union Medical College, Chinese Academy of Medical Sciences, Beijing, 100193 China; 3 Dazhou Academy of Agricultural Sciences, Dazhou, 635000 China; 4 Tianjin University of Traditional Chinese Medicine, Tianjin, 10063 China

**Keywords:** Phosphorus deficiency, *Epimedium pubescens*, Transcriptome analysis, Flavonoid biosynthesis

## Abstract

Phosphorus, a crucial macronutrient essential for plant growth and development. Due to widespread phosphorus deficiency in soils, phosphorus deficiency stress has become one of the major abiotic stresses that plants encounter. Despite the evolution of adaptive mechanisms in plants to address phosphorus deficiency, the specific strategies employed by species such as *Epimedium pubescens* remain elusive. Therefore, this study observed the changes in the growth, physiological reponses, and active components accumulation in *E. pubescens*under phosphorus deficiency treatment, and integrated transcriptome and miRNA analysis, so as to offer comprehensive insights into the adaptive mechanisms employed by *E. pubescens* in response to phosphorus deficiency across various stages of phosphorus treatment. Remarkably, our findings indicate that phosphorus deficiency induces root growth stimulation in *E. pubescens*, while concurrently inhibiting the growth of leaves, which are of medicinal value. Surprisingly, this stressful condition results in an augmented accumulation of active components in the leaves. During the early stages (30 days), leaves respond by upregulating genes associated with carbon metabolism, flavonoid biosynthesis, and hormone signaling. This adaptive response facilitates energy production, ROS scavenging, and morphological adjustments to cope with short-term phosphorus deficiency and sustain its growth. As time progresses (90 days), the expression of genes related to phosphorus cycling and recycling in leaves is upregulated, and transcriptional and post-transcriptional regulation (miRNA regulation and protein modification) is enhanced. Simultaneously, plant growth is further suppressed, and it gradually begins to discard and decompose leaves to resist the challenges of long-term phosphorus deficiency stress and sustain survival. In conclusion, our study deeply and comprehensively reveals adaptive strategies utilized by *E. pubescens* in response to phosphorus deficiency, demonstrating its resilience and thriving potential under stressful conditions. Furthermore, it provides valuable information on potential target genes for the cultivation of E. pubescens genotypes tolerant to low phosphorus.

**Supplementary Information:**

The online version contains supplementary material available at 10.1186/s12870-024-05063-y.

## Introduction

Phosphorus stands as a pivotal nutrient influencing plant physiological metabolism, assuming a crucial regulatory role in processes such as photosynthesis, DNA and RNA synthesis, protein synthesis, enzyme activity regulation, gene regulation, signal transduction, among others. Approximately 43% of the world’s cultivated land faces phosphorus deficiency [1], primarily due to the propensity of inorganic phosphates (Pi) available to plants to form insoluble complexes with cations like Fe and Ca in the soil [2, 3], consequently diminishing available phosphorus content. Consequently, phosphorus deficiency emerges as a prevalent stress encountered by plants, impacting diverse physiological and metabolic processes. These include the accumulation of reactive oxygen species, impairment of intracellular structures (e.g., chloroplasts), disruption of electron chain transmission, inhibition of photosynthesis, reduction in ATP production, and interference with carbon assimilation, collectively impeding plant growth and development [4–6]. Thus, comprehending the coping mechanisms of medicinal plants under phosphorus deficiency proves crucial for enhancing medicinal material yield, developing new varieties with heightened phosphorus use efficiency, and promoting soil ecosystem health.

When plants confront phosphorus deficiency stress, they undergo adaptive changes across phenotypic, physiological, biochemical, and molecular levels [7, 8]. In response to phosphorus deficiency, plants adjust their root-to-shoot ratio [9], optimize root structure, and produce root exudates [10] to enhance phosphorus absorption efficiency. Meanwhile, photosynthetic-related parts are significantly suppressed, and physiological metabolic pathways are adjusted to optimize internal phosphorus cycling [11, 12] or tolerate phosphorus deficiency. Changes in transcription levels are the earliest response of plants to stress, regulating phenotypic and metabolic pathway changes accordingly. Integrated transcriptomics analysis can reveal the response mechanisms of plants to phosphorus deficiency stress at a deeper level. Changes in transcription levels are the first response of plants to stress, regulating phenotypic and metabolic pathway changes accordingly. Integrated transcriptomics analysis can reveal the response mechanisms of plants to phosphorus deficiency stress at a deeper level. Under phosphorus deficiency stress, the expression of genes encoding key enzymes in the phenolic and flavonoid metabolic pathways in the leaves of Artemisia argyi is upregulated, consistent with the significant increase in the content of phenolic and flavonoid compounds in the leaves [13]. Peanut differentially expressed multiple hormone-related genes, antioxidant enzyme-related genes, and phosphate transporter genes in leaves and roots, thereby affecting hormone levels and antioxidant enzyme activity changes, and improving internal phosphorus cycling [14]. Under phosphorus deficiency conditions, soybeans respond by increasing the expression of the *GmPAP* gene family in their roots, which facilitates the symbiosis with rhizobia or arbuscular mycorrhizae, thus promoting phosphorus absorption in the root system [15]. Cotton adjusts its internal phosphorus cycling, root-to-shoot growth changes, and stress resistance by upregulating the expression levels of genes related to phosphorus metabolism, carbohydrate metabolism, and flavonoid metabolism in response to phosphorus deficiency stress [16]. Additionally, plant microRNAs (miRNAs) regulate plant growth and development under stress conditions through the cleavage of target gene mRNAs for post-transcriptional inhibition [17]. For example, miR399 influences phosphorus metabolism by downregulating the expression of its target gene PHO2 (phosphate starvation response gene PHO2), affecting phosphorus absorption and release [18]. Under phosphorus-deficient conditions, peanuts regulate their response to phosphorus stress through post-transcriptional regulation mediated by miRNAs, which affect hormone signal transduction and synthesis, as well as lignin synthesis pathways [14, 19]. Consequently, the multilevel changes in gene expression reflect the genetic adaptability of plants responding to phosphorus limitation, underscoring the heightened sensitivity of plants to environmental stress. This provides a critical molecular mechanism for the adaptive evolution of plants under nutrient-limited conditions. However, different plant species, tissue types, and growth stages often employ diverse strategies in response to phosphorus deficiency stress. Therefore, understanding their reaction mechanisms and response changes to phosphorus deficiency stress, proving to be crucial, for designing effective improvement strategies to enhance phosphorus absorption and utilization efficiency or resistance to phosphorus deficiency stress in specific plants [20, 21].*Epimedium pubescens*, known as Yin Yang Huo in traditional Chinese medicine for nearly 2000 years [22], holds significant medicinal value within the Berberidaceae family. The leaves of this plant house prenylated flavonol glycosides (PFGs) with diverse pharmacological activities, including antioxidation, immune system enhancement, and cardiovascular and neuroprotective effects [22, 23]. Notably, four Icariin-flavonoids (Epimedin A, Epimedin B, Epimedin C, and Icariin) serve as crucial bioactive markers for assessing the quality of PFGs [22, 24].

Research has indicated that the accumulation of flavonoids in plants can be influenced by phosphorus deficiency, showcasing variations in different plant parts and species. For instance, while phosphorus deficiency increases flavonoid metabolites in the roots of *Arabidopsis* and soybean [25, 26], the response varies in tea plants, where leaves accumulate anthocyanins, roots accumulate proanthocyanidins, and buds show changes in chalcones [27]. Understanding how phosphorus deficiency affects the accumulation of bioactive PFGs, especially the four Icariin-flavonoids in *Epimedium pubescens*, presents an intriguing avenue for investigating the adaptive mechanisms of this plant to phosphorus stress.

A soil investigation in the primary production areas of *E. pubescens* revealed a common challenge of low available phosphorus content (10–20 mg/kg). Despite the acknowledged role of phosphorus fertilizer in Epimedium cultivation, a systematic examination of the adaptive physiological changes and underlying molecular mechanisms of flavonoid metabolism under phosphorus deficiency is lacking. The assembly and analysis of *E. pubescens* genome [28], along with time-series transcriptome analysis during leaf development, have provided initial insights [29]. However, the response mechanism of *E. pubescens* to phosphorus deficiency stress remains elusive, based on existing literature research, we hypothesize that the molecular mechanisms of *Epimedium pubescens* in responding to phosphorus deficiency stress primarily involve the regulation of carbon metabolism, phosphorus metabolism and transport, hormone signaling, flavonoid synthesis pathways, and post-transcriptional regulation mediated by miRNAs. Ultimately, these molecular changes manifest as phenotypic and physiological modifications. This study aims to bridge this gap by investigating phenotypic and physiological changes under phosphorus deficiency and normal phosphorus application treatments over different time scales. The integration of transcriptome analysis with miRNA sequencing will facilitate a comprehensive examination of physiological, biochemical, and Icariin-flavonoid component changes. The ultimate goal is to furnish reliable scientific evidence for optimizing phosphorus absorption and utilization strategies in *E. pubescens*, enhancing the quality of medicinal materials, and developing phosphorus-efficient varieties. This research is poised to contribute valuable knowledge to the field of medicinal plant cultivation and sustainable utilization.

## Materials and methods

### Plant materials and treatments

In October 2022, one-year-old, healthy seedlings of *Epimedium pubescens* with similar growth patterns (having 5–7 simple leaves and 1–3 trifoliate leaves) were collected from cultivation bases in Dazhou City, Sichuan Province (31°28′19.90″N, 107°39′8.37″E). These seedlings were identified as *Epimedium pubescens*, a species belonging to the genus *Epimedium* in the family Berberidaceae, by Professor Baolin Guo from the Institute of Medicinal Plant Development, Chinese Academy of Medical Sciences. Voucher specimens of these seedlings are preserved in the Herbarium of the Institute of Medicinal Plant Resources Development, Chinese Academy of Medical Sciences, with the preservation number B.L.Guo 0711-3. The seedlings were then transplanted into sand culture pots with a substrate ratio of river sand to perlite (3:2). Initially, the seedlings underwent a 20-day acclimation period in a 12.5% Hoagland nutrient solution, with applications administered every 10 days. Once the seedlings developed new roots and resumed normal growth, they were subjected to two treatments: phosphorus deficiency (-P, 0 mmol/L Pi) and normal phosphorus (+ P, 1 mmol/L Pi). The composition and concentrations of nutrients are detailed in Table S1. The pH of the nutrient solution was adjusted to 6.5, and applications were administered every two weeks. When watering each time, pour 200 milliliters of water into each plant (ensuring that there is a uniform overflow of solution from the bottom of the pot so that all the roots of the plant are submerged in the nutrient solution). Each treatment was replicated three times, with 12 seedlings per replication.

The experimental seedlings were placed in a controlled environmental plant growth chamber with a light intensity of 2000 lx (14 h·d^− 1^) and a temperature maintained at 22 ± 2 °C. After treatment, we collected fresh leaves from plants at both the early stage of phosphorus deficiency stress (30 days), when no obvious damage symptoms were observed, and the late stage of phosphorus deficiency stress (90 days), when obvious damage symptoms appeared. The leaves were rapidly frozen with liquid nitrogen and stored at -80℃ for future testing. Additionally, we measured relevant indicators and harvested the plants on the 90th day.

### Measurement of growth indices

After 90 days of cultivation under both treatments (-P and + P), the phenotypic characteristics of *E. pubescens* were evaluated. The number of leaves was recorded, and leaf area was measured using a leaf area meter. Samples of the roots, rhizomes, stems, and leaves were collected from each plant and dried in an oven at 45 °C until a constant weight was achieved. The dry weights of each part were then recorded (three replicates per treatment).

### Measurement of photosynthetic parameters and chlorophyll content

On the 90th day of the experiment, between 8:00 and 11:00 AM, the photosynthetic rate of functional leaves from similar positions within each treatment group, specifically the second compound leaf counted from the top of the rhizome downward, was measured using a LI-6400 Portable Photosynthesis System (LI-COR, USA). The chlorophyll content was measured using a chlorophyll meter SPAD-502 (Konica Minolta Inc, Japan) by calculating the SPAD value (relative chlorophyll content) of the leaves based on the ratio of light intensity without clamping the leaves and the transmission light ratio after clamping the leaves at wavelengths of 650 and 940 nm.

### Scanning and analysis of root system structure

Following the 90-day treatment period, indices such as total root length, root diameter, root volume, and surface area were measured for the plants to analyze the effects of two phosphorus levels (-P and + P) on the phenotypic characteristics of the plant root system. Fresh roots were scanned using an Epson Perfection V850 Pro photo scanner, and the scanned images were analyzed using WinSEEDLE Pro 2020a and WinRHIZO Pro 2015 software (Regent Instructions, Canada Inc.).

### Measurement of phosphorus content

Weigh 0.5 g of dried *E. pubescens* leaf powder into a digestion tube. Digest the sample using sulfuric acid-hydrogen peroxide. The total phosphorus content is then determined using the molybdenum-antimony anti-colorimetry method [30].

### Measurement of Icariin-flavonoids content

Following the freeze-drying of fresh *E. pubescens* leaves stored at -80 °C, the Icariin-flavonoids content was determined using ultra-high-performance liquid chromatography (UPLC). A total of 50 milligrams of freeze-dried leaf powder was extracted with 5 milliliters of 50% ethanol using ultrasonication for 30 min. Subsequently, the extract was filtered through a 0.22 μm filter membrane. Chromatographic analysis was conducted using an ACQUITYTM UPLC system (Waters, USA) equipped with an ACQUITY UPLCR BEH C18 column (2.1 mm × 100 mm, 1.7 μm). The mobile phase consisted of solvent A (water) and solvent B (acetonitrile). The gradient elution program was set as follows: 0 min, 79% A; 6 min, 71% A; 12 min, 56% A; 17 min, 5% A.

### RNA extraction from leaf samples and transcriptome sequencing

Total RNA extraction from plant leaves treated with -P (0 mmol/L Pi) and + P (1 mmol/L Pi) was performed using the RNAprep Pure Polysaccharide/Polyphenol Plant Total RNA Extraction Kit (QIAGEN, Germany). The quality and integrity of the RNA were assessed using the Agilent 2100 Bioanalyzer (Agilent Technologies, USA). For RNA sequencing (RNA-seq) library preparation, three biological replicates were sequenced for each sample using the Illumina NovaSeq 6000 sequencing platform (Illumina, USA). Gene expression levels were normalized using Fragments Per Kilobase Million (FPKM) to control for sequencing depth and transcript length.

HISAT2 v2.0.5 was employed to align the sequencing data to the reference genome (*Epimedium pubescens* [28]), and Stringtie 1.3.3b [31] was used to identify known gene transcripts and predict novel gene transcripts. Differential gene expression between treatments was compared using the DESeq2 software (1.20.0). The resulting *P*-values were adjusted using the Benjamini & Hochberg method to control the false discovery rate, and genes with a *p*-adj < 0.05 and |log_2_FC| ≥ 1 were considered as differentially expressed genes (DEGs). The clusterProfiler (3.8.1) software was employed to conduct GO and KEGG enrichment analysis on the DEGs.

### miRNA expression analysis

For miRNA expression profiling, the same samples as those used for transcriptome analysis were then subjected to miRNA sequencing. Library preparation was performed using the NEB Next® Multiplex Small RNA Library Prep Set for Illumina® (NEB E7300L). Expression levels were normalized using the TPM (Transcripts Per Million) method [32]. Differentially expressed miRNAs across treatments were analyzed with DeSeq2 software, applying the criteria of *p*-val < 0.05 and |log_2_FC|≥ 1. The characteristic hairpin structure of miRNA precursors was employed for the prediction of new miRNAs, integrating the miRNA prediction software miREvo [33] and mirdeep2 [34]. To scrutinize the functional response of miRNAs under phosphorus deficiency stress in *E. pubescens* leaves, psRobot (http://omicslab.genetics.ac.cn/psRobot/) was utilized for predicting target genes of the miRNAs. The evaluation and screening criterion relied on sequence complementarity between the target mRNAs and mature miRNAs [35]. Differential miRNAs were juxtaposed with transcriptome data to discern target mRNAs, eliminating those exhibiting similar expression trends to the miRNAs and those encoding uncharacterized proteins.

### Quantitative real-time PCR (qRT-PCR) validation

Validation of Transcriptome Sequencing: The extracted RNA was then reverse transcribed into cDNA using the Reverse Transcriptase Kit (M-MLV) (Beijing Zoman Biotechnology Co., Ltd., China). Nine genes were selected for validation, with β-Actin-1 serving as the internal reference gene. Primer sequences were designed using Primer Premier 6.0 software, and the detailed genes and primer sequences are provided in Table S2. qRT-PCR validation was conducted using the 2xHQ SYBR qPCR Mix (Without ROx), (Beijing Zoman Biotechnology Co., Ltd, China). Each gene underwent three technical replicates, and each treatment sample involved three biological replicates. The relative gene expression was calculated using the 2^−ΔΔCt^ method to confirm the reliability of the transcriptome.

Validation of miRNA High-throughput Sequencing: Five microRNAs and five target genes were selected for qRT-PCR validation. Reverse transcription was performed using the miRNA First Strand cDNA Synthesis Tailing Reaction Kit (Sangon Biotech, Shanghai, China) according to the manufacturer’s instructions. qRT-PCR reactions were conducted using the MicroRNAs qPCR SYBR Green Method Kit (Sangon Biotech, Shanghai, China). U6 was used as the reference gene, and the primer information is provided in Table S2. The relative expression levels of target genes were detected using the same method described in the transcriptome validation.

### Statistical analysis

A completely randomized design (CRD) was adopted for all tests conducted, with three repetitions. SPSS 26.0 was used for statistical analysis. To compare differences between two treatments, the independent T-test was applied. For comparing differences among various treatments at different time points, the Duncan method (also known as the new multiple range method) was employed. Significance was established at *p* < 0.05.

## Results and analysis

### Effects of phosphorus deficiency stress on the growth and phosphorus content of *E. pubescens*

**Fig. 1:**
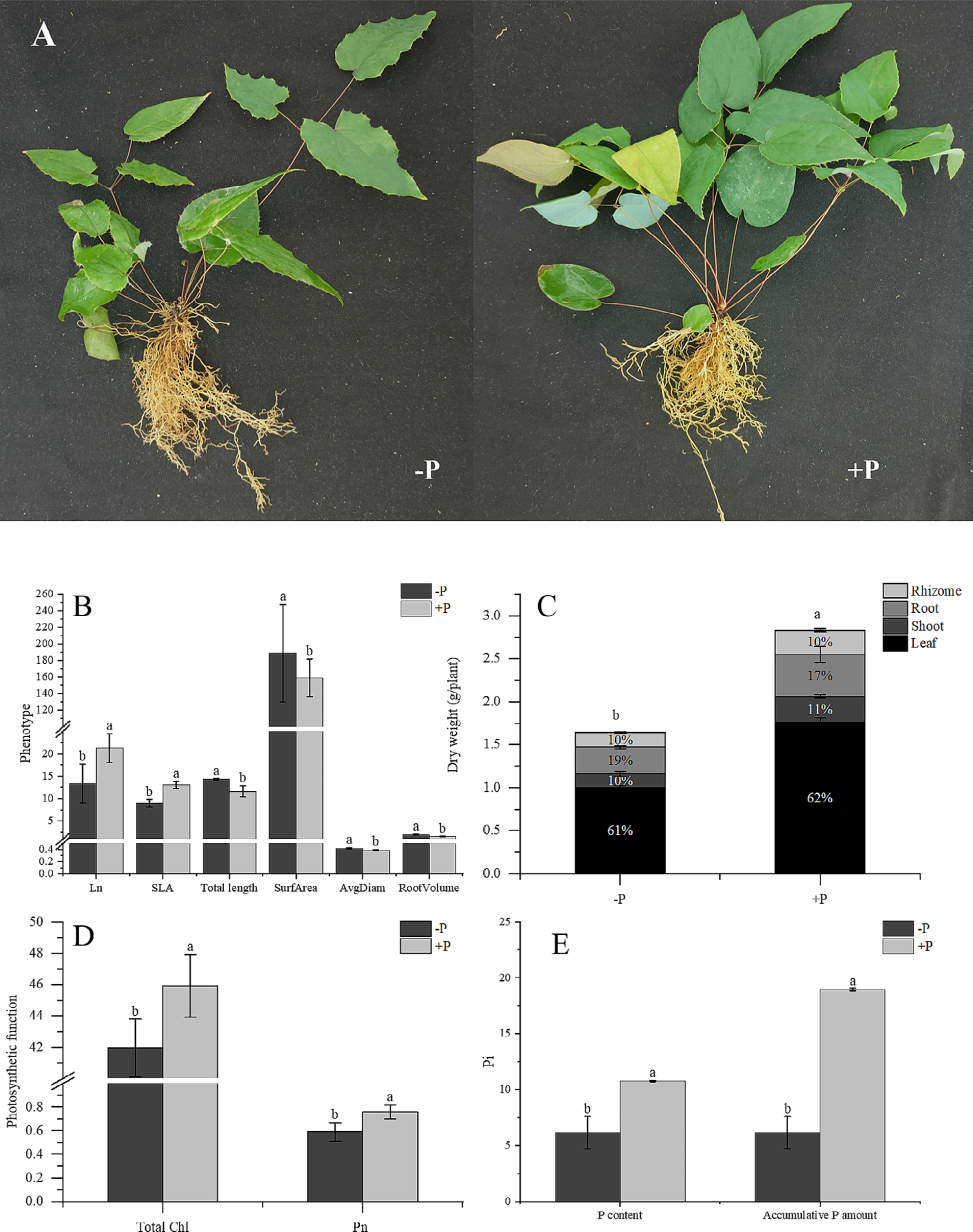
Changes in *E. pubescens* Plants after 90 Days of Two Phosphorus Treatments: (**A**) Morphological Differences between Phosphorus Treatments; (**B**) Phenotypic Indices, Ln: Leaf Number (n), SLA: Specific Leaf Area (cm^2^), Total Length: Total Root Length (cm), Surf Area: Root Surface Area (cm^2^), AvgDiam: Average Root Diameter (mm), RootVolume: Root Volume (cm^3^); (**C**) Total Biomass and the Proportion of Biomass in Each Plant Part under Two Phosphorus Treatments; (**D**) Photosynthesis-Related Indices. Total Chl: Relative Chlorophyll Content (SPAD), Pn: Photosynthetic Rate (μmolCO_2_·m^− 2^· S^− 1^), (**E**) Leaf Phosphorus Content (mg/g) and Phosphorus Accumulation (mg). Different letters indicate significant differences (*p* < 0.05)

Significant differences in the growth and development of the plants were observed after the 90-day treatment with the two phosphorus levels. Notably, phosphorus deficiency stress had a profound impact on the overall morphogenesis of the plants, significantly hindering biomass accumulation in *E. pubescens*. Visually, plants treated with -P exhibited chlorophyll deficiency, sparse leaves, and a sparse and elongated root system (Fig. 1A). Specifically, in comparison to the + P treatment, the -P treatment showed a marked reduction in leaf number and leaf area, constituting merely 62% and 69% of the corresponding values in the + P treatment, respectively. However, the -P treatment resulted in significantly higher total root length, root surface area, root diameter, and root volume compared to the + P treatment (Fig. 1B). Additionally, the -P treatment markedly reduced the biomass of various plant parts. The dry weights of leaves, stems, roots, and rhizomes decreased by 47%, 47%, 37%, and 39%, respectively, in comparison to the + P treatment, leading to a 42% decrease in total plant biomass (Fig. 1C).

Plant growth and development are intricately linked to the efficiency of photosynthesis. Therefore, assessing the photosynthetic rate of *E. pubescens* leaves proves valuable in elucidating variations in plant growth under different phosphorus concentrations. In comparison to the + P treatment, the -P treatment results in a significant reduction in the relative chlorophyll content (SPAD) and photosynthetic rate of the plants, aligning with the observed trends in plant phenotype and biomass changes (Fig. 1D). This suggests that under phosphorus deficiency stress, the diminished photosynthesis leads to a reduction in plant carbon assimilation. Consequently, plants adapt by reallocating carbon from leaves to roots, augmenting the absorption area of the roots in the soil. This strategic adjustment aids the plants in more effectively absorbing limited Pi resources, thereby facilitating adaptation to phosphorus-deficient environments.

In addition, further tests on leaf Pi content revealed that the Pi content and Pi accumulation (Pi content × leaf dry weight) in leaves under -P treatment were significantly lower than those under + P treatment, accounting for only 43% and 68% of the + P treatment (Fig. 1E). This indicates that phosphorus deficiency treatment significantly reduced the Pi content and Pi accumulation in leaves, inhibited physiological processes such as photosynthesis, and biomass accumulation, thereby affecting plant growth and development.

### Effect of phosphorus deficiency stress on Icariin-flavonoid accumulation

**Fig. 2:**
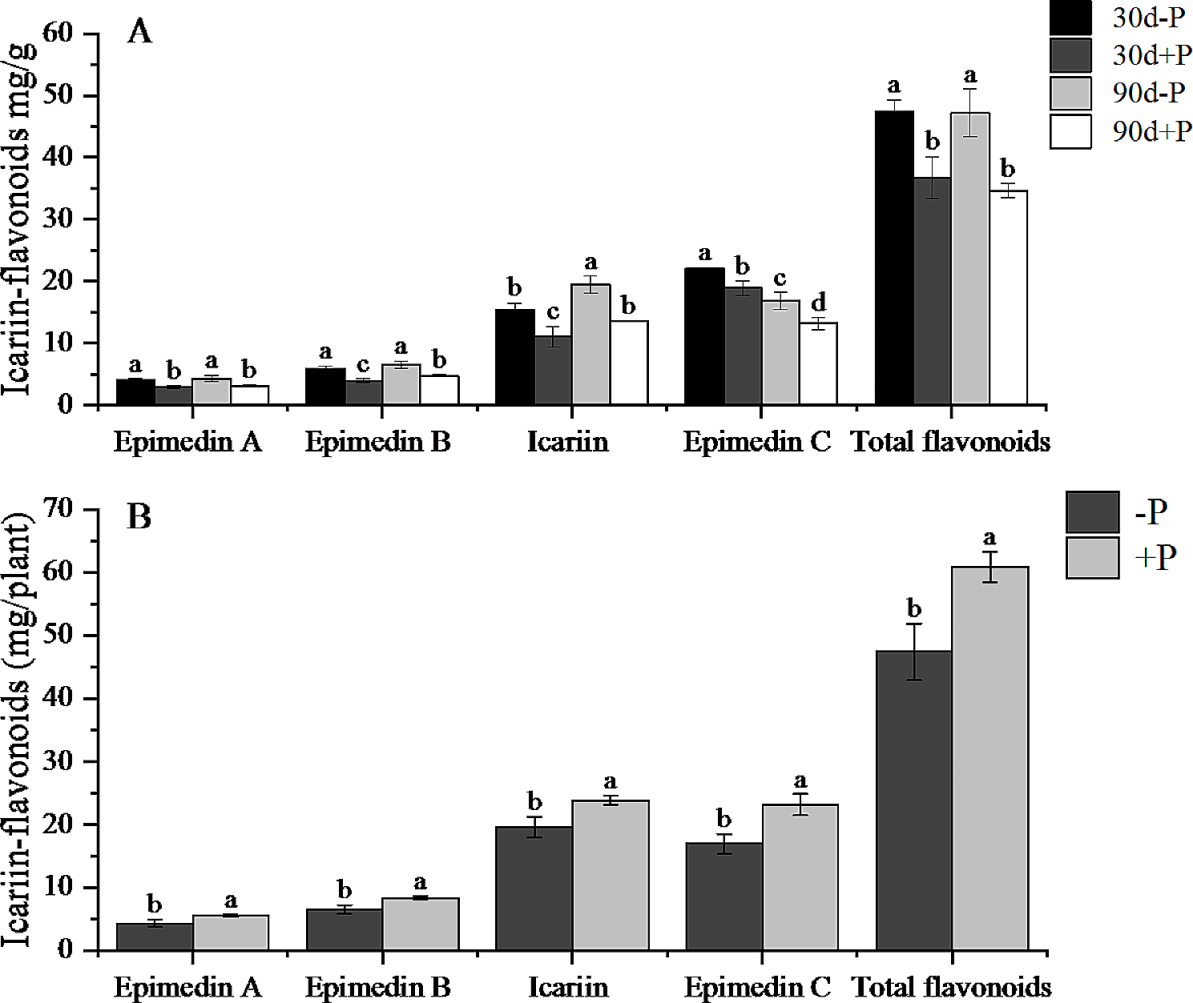
(**A**) Icariin-flavonoids content (mg/g) in *E. pubescens* leaves under different phosphorus levels at 30d and 90d. (**B**) Total production of Icariin-flavonoids (mg/plant) in individual leaves of *E. pubescens* under different phosphorus levels. Different letters indicate significant differences (*p* < 0.05)

Prior research has highlighted that phosphorus deficiency can stimulate the accumulation of flavonoids in leaves or roots, associated with their antioxidant function or the recruitment of beneficial microorganisms [25–27]. In our investigation, substantial variations were observed in the levels of Icariin-flavonoids (Epimedin A, Epimedin B, Epimedin C, Icariin) and total flavonoids in *E. pubescens* leaves under the -P treatment compared to the + P treatment, exhibiting significant increases at both the 30th and 90th days. However, a closer examination of the yield per plant, obtained by multiplying the dry weight of individual plant leaves with the flavonoid content, revealed a notable reduction in the final yield of Icariin-flavonoids and total flavonoids per plant under the -P treatment in comparison to the + P treatment (Fig. 2A). This suggests that phosphorus deficiency promotes the accumulation of Icariin-flavonoids in the leaves of *E. pubescens*. Possibly, *E. pubescens* adapts to phosphorus deficiency by elevating the content of Icariin-flavonoids to counteract Reactive Oxygen Species (ROS) within the leaves. However, the severe limitation in the plant’s biomass accumulation concurrently results in a decline in the final yield (Fig. 2B).

Compared to the content at 30 days, the content of Epimedin C in leaves significantly increased at 90 days, while the content of Icariin significantly decreased (Fig. 2A). This is due to the accumulation and variation of flavonoid glycosides that occur during the growth and development of *E. pubescens* leaves [29].

### Transcriptome analysis of leaves at different stages

In this study, transcriptome analysis was conducted on *E. pubescens* leaves subjected to distinct phosphorus levels at 30 days (30d) and 90 days (90d). The sequencing process generated a total of 80.42 gigabytes (GB) of high-quality clean data, exhibiting a Q30 score of 94.19% and an average GC content of 44.82%. These metrics ensure the reliability and precision of the sequencing results. Alignment of the clean data from each sample with the *E. pubescens* reference genome achieved a robust alignment rate ranging from 87.84 to 89.89%.

#### Transcriptional changes in *E. Pubescens* leaves under phosphorus deficiency stress at 30d

Screening for differentially expressed genes (DEGs) in *E. pubescens* leaves subjected to distinct phosphorus treatments at 30 days (30d) was performed using the criteria of *p*-adj < = 0.05 and |log_2_FC| >= 1.0. This analysis identified a total of 564 DEGs. Compared to the 30d + P treatment, the 30d -P treatment exhibited significant upregulation of 222 DEGs and downregulation of 342 DEGs (Fig. S1A).

Functional analysis utilizing Gene Ontology (GO) and Kyoto Encyclopedia of Genes and Genomes (KEGG) pathways provided insights into the biological implications of these DEGs. In the 30d -P treatment, upregulated GO categories encompassed processes like ‘polysaccharide metabolic process’, ‘cellular glucan metabolic process’, and ‘carbohydrate catabolic process’, whereas ‘cellulose biosynthetic process’, ‘polysaccharide biosynthetic process’, and ‘photosystem II oxygen evolving complex’ were downregulated. KEGG enrichment highlighted the upregulation of pathways such as ‘Plant hormone signal transduction’, ‘Flavonoid biosynthesis’, and ‘Plant-pathogen interaction’, alongside the downregulation of pathways like ‘Biosynthesis of nucleotide sugars’, ‘Steroid biosynthesis’, and ‘Phenylpropanoid biosynthesis’ in the 30d -P treatment (Table S3).

Remarkably, the observed upregulation in the ‘Flavonoid biosynthesis’ pathway aligns well with the UPLC detection results (Fig. 2). This correspondence suggests the upregulation of structural genes linked to Icariin-flavonoids synthesis in leaves under phosphorus deficiency treatment, potentially promoting the accumulation of Icariin-flavonoids, including Epimedin A, Epimedin B, Epimedin C, and Icariin. Flavonoids, constituting a diverse class of secondary metabolites derived from phenylalanine, have a well-elucidated biosynthetic pathway [36, 37]. To investigate further the molecular regulatory mechanism of the Icariin-flavonoids biosynthesis pathway under phosphorus deficiency stress, we conducted an analysis of the enriched flavonoid biosynthesis pathway using KEGG through the localized Gene Set Enrichment Analysis (GSEA) software (http://www.broadinstitute.org/gsea/index.jsp). In contrast to conventional differential gene analysis, GSEA enrichment analysis transcends individual genes, concentrating on gene set enrichment. This approach theoretically facilitates the capture of subtle yet coordinated changes influencing biological pathways and helps avoid gene loss [38].

**Fig. 3:**
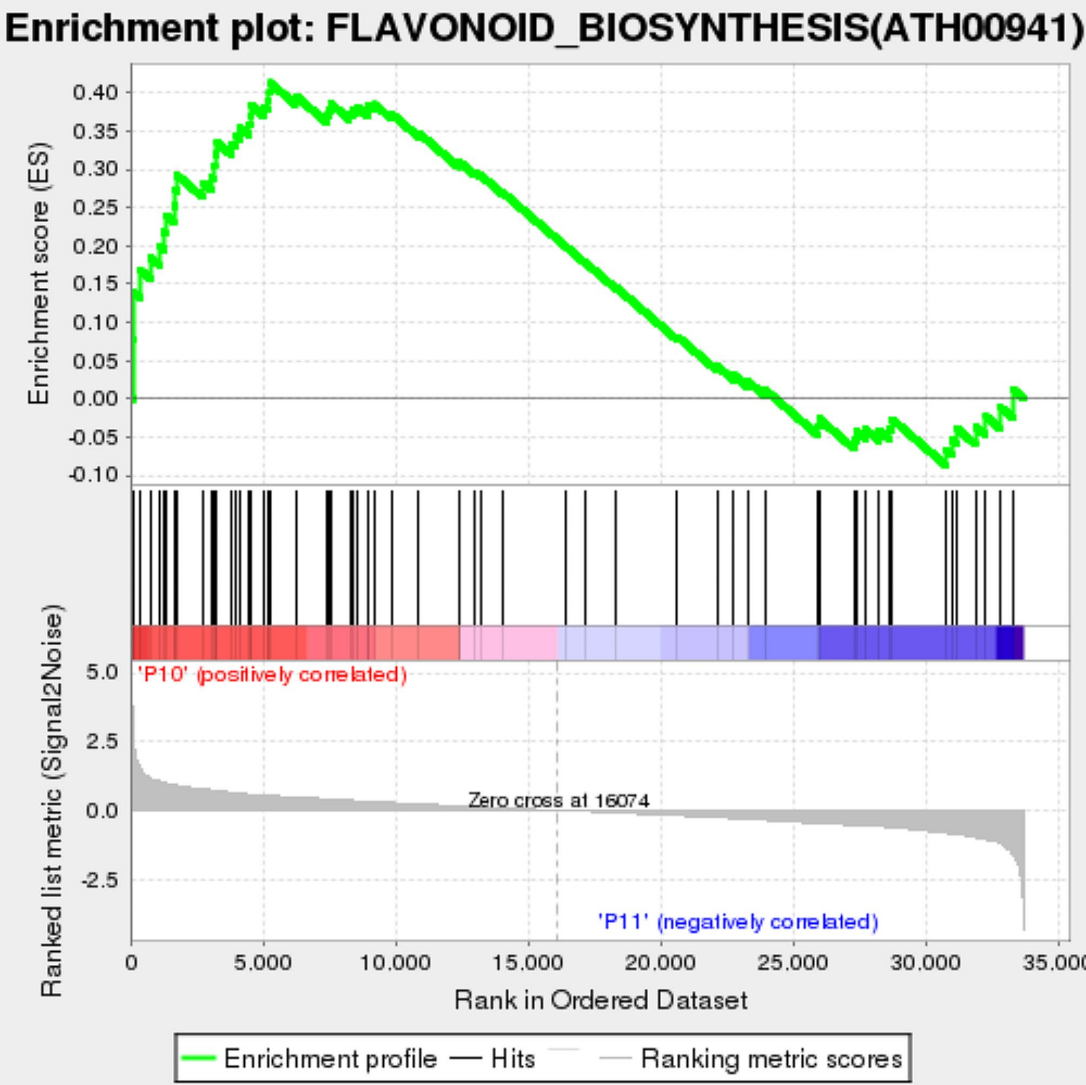
Enrichment plot: FLAVONOID_BIOSYNTHESIS(ATH00941). Profile of the Running ES Score & Positions of GeneSet Members on the Rank Ordered List (P10: 30d -P treatment, P11: 30d + P treatment)

**Fig. 4:**
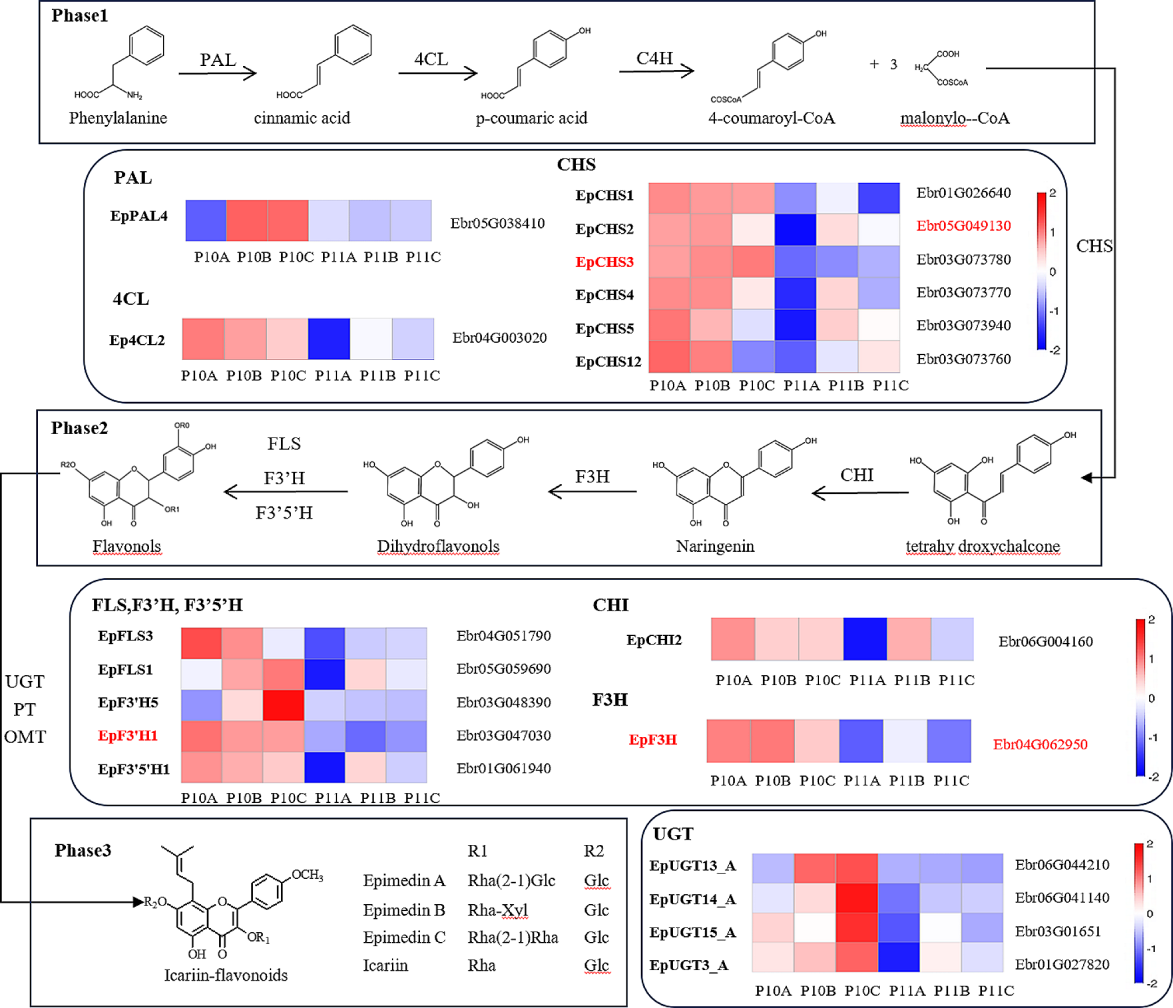
Changes in gene expression related to Icariin-flavonoids biosynthesis in *E. pubescens* leaves under phosphorus deficiency stress for 30 days (The gene annotations are bold on the left side of the heatmap, and gene IDs are on the right. P10: 30d -P treatment, P11: 30d + P treatment. A, B, C represent different replicates)

The peak value and shape of the green curve in Fig. 3 reflect the enrichment of the gene set in the sorted list. The peak of ES is reached in the P10 sample (30d -P treatment), indicating that this pathway is upregulated in the P10 sample. The section from (0,0) on the x-axis to the location where the ES peak appears is the Leading-edge subset, which is closely related to phenotypic changes. The genes in this subset have a value of “YES” in the “CORE ENRICH” column of Table S4, so we selected these genes for analysis. The black vertical bars in the Hits plot below indicate the distribution of genes, while the Ranking metric scores plot shows the distribution of rank values for all genes after sorting. Utilizing genes enriched by GSEA (Fig. 3, Table S4) and integrating them with known key structural genes participating in Icariin-flavonoids biosynthesis in *E. pubescens* leaves [29], we constructed a schematic diagram delineating the Icariin-flavonoids biosynthetic pathway in *E. pubescens* leaves under 30 days of phosphorus deficiency stress (Fig. 4). In Phase 1, PAL, C4H, and 4CL act collaboratively to convert phenylalanine to p-coumaroyl-CoA. Moving to Phase 2, CHS and CHI catalyze the transformation of p-coumaroyl-CoA into chalcone, a precursor for flavonol and flavone biosynthesis. The upregulation of genes such as F3’5’H, F3H, F3’H, and FLS facilitates flavonol synthesis. Finally, Phase 3 involves genes like UGT, PT, and OMT, driving the formation of prenyl and methoxy groups on flavonols, culminating in the conjugation of a monosaccharide to form Icariin-flavonoids.

**Fig. 5:**
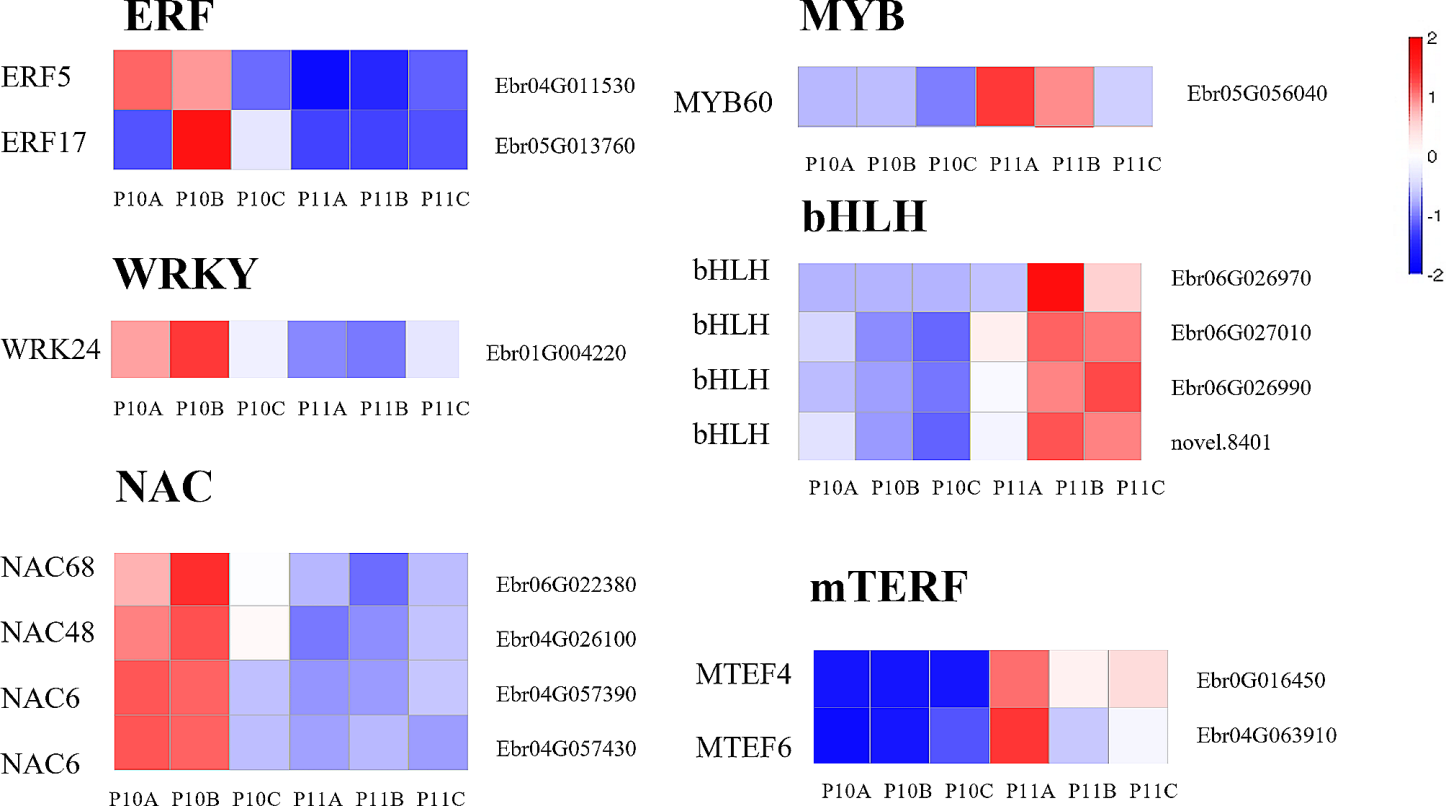
Heatmap of differential TF family expression in *E. pubescens* leaves under phosphorus deficiency stress for 30 days (The gene annotations are on the left side of the heatmap, and gene IDs are on the right. P10: 30d -P, P11: 30d + P. A, B, C are different duplicates). The |log_2_FC| is represented by colors

Additionally, transcription factors regulate the expression of downstream genes through mechanisms such as splicing or silencing. Of the DEGs, 27 transcription factors were identified, belonging to 18 families. Families including WRKY, NAC, ERF, bHLH, and MYB are closely associated with plant stress response [39]. Therefore, we focused on the changes in these TF families under phosphorus deficiency stress. The results indicated that WRKY24, NAC68, NAC48, NAC6, ERF5, and ERF17 genes were up-regulated, while MYB60 and members of the bHLH family were down-regulated in the 30d -P treatment. Furthermore, members of the mTERE family related to chloroplast formation (MTEF4, MTEF6) were down-regulated in the 30d -P treatment (Fig. 5).

#### Transcriptional changes in *E. Pubescens* leaves under phosphorus deficiency stress at 90d

Screening for differentially expressed genes (DEGs) in the leaves of *E. pubescens* under different phosphorus levels for 90 days resulted in a total of 173 DEGs. Compared to the 90d + P treatment, 47 DEGs were significantly upregulated, while 126 DEGs were downregulated in the -P treatment (Fig. S1B). Functional analysis of these DEGs was conducted using GO and KEGG. The GO enrichment results showed that the GO categories of ‘pigment metabolic process’, ‘sulfate transport’, ‘nuclear envelope’, and ‘polygalacturonase activity’ were upregulated in the 90d-P treatment, while the GO categories of ‘ER to Golgi vesicle-mediated transport’ and ‘iron-sulfur cluster binding’ were downregulated. The KEGG enrichment results indicated that the pathways of ‘Purine metabolism’, ‘Starch and sucrose metabolism’, and ‘Porphyrin metabolism’ were upregulated in the 90d-P treatment, while the pathways of ‘Steroid biosynthesis’, ‘Protein processing in endoplasmic reticulum’, and ‘Biosynthesis of various plant secondary metabolites’ were downregulated (Table S5).

**Fig. 6:**
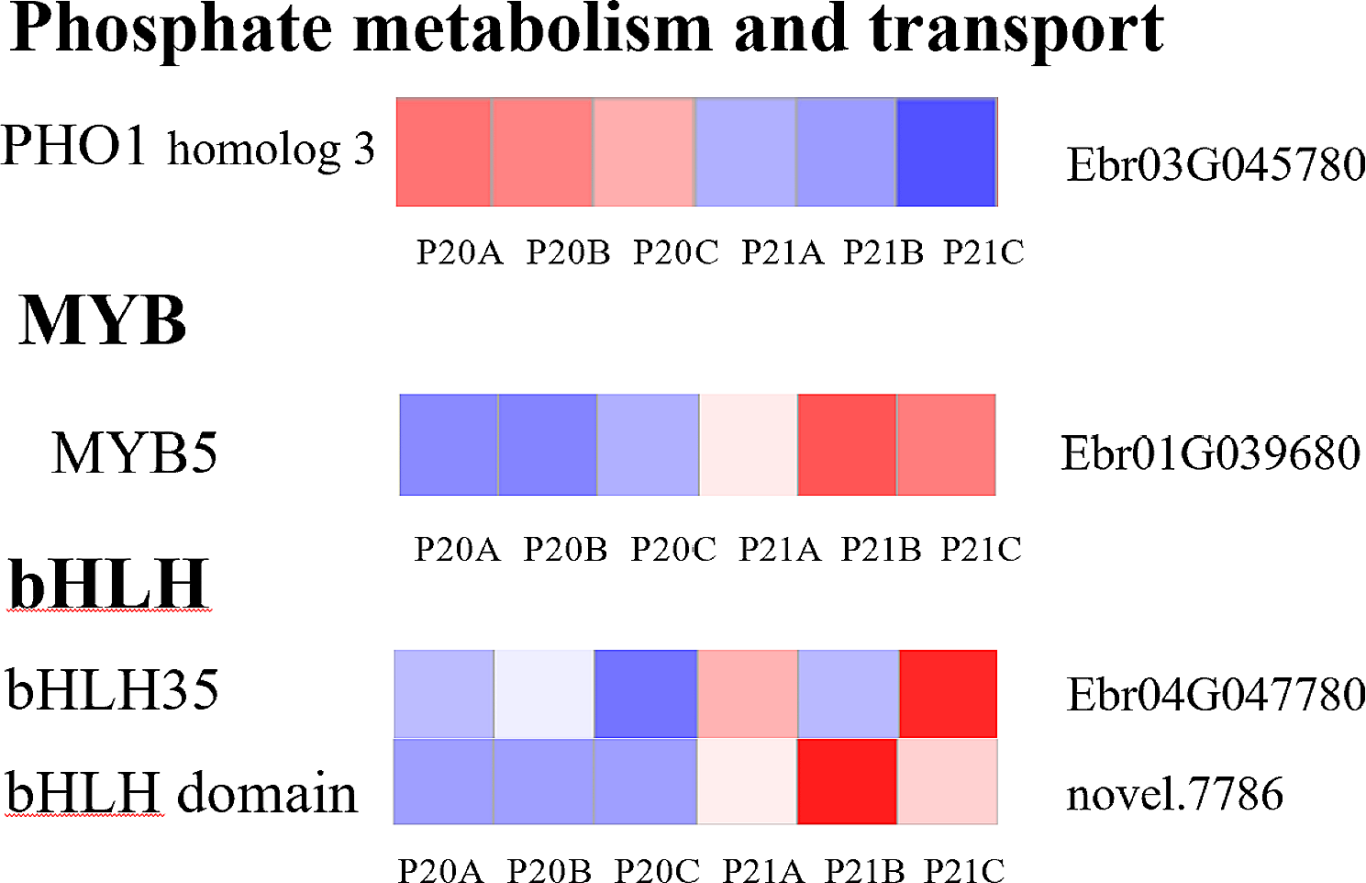
Heatmap of DEGs (*p*-val < 0.05) in *E. pubescens* leaves under phosphorus deficiency stress for 90 days compared to normal phosphorus conditions. Heatmaps of phosphorus metabolism and transport-related DEGs, as well as major TF families, are also shown (gene annotations are on the left side of the heatmap, gene IDs are on the right, P20: 90d -P treatment, P21: 90d + P treatment, A, B, C represent different duplicates). The |log_2_FC| is represented by colors

PHT and SPX domain-containing proteins play crucial roles in maintaining phosphorus homeostasis and fine-tuning phosphorus transport signaling [40, 41]. Under 90d-P treatment, the gene encoding phosphate transporter (PHO1 homolog 3) is upregulated (Fig. 6). Furthermore, the commonly observed stress-responsive transcription factor families MYB and bHLH [39] were also identified (Fig. 6).

The number of DEGs identified between the 90d -P and + P treatments is relatively small, resulting in limited transcriptional information available. This may be due to the strict screening criteria for DEGs and insufficient annotation of the transcriptome. Adjusting these criteria may yield more transcriptional information, but the reliability of this information would also be affected. Based on the criterion of *p*-value < 0.05, GO and KEGG enrichment analyses were conducted separately for upregulated and downregulated genes among the obtained DEGs (Table S6). Additionally, genes encoding phosphate transporters (PHT1-4), proteins with SPX domains (SPX1, SPXM3), and purple acid phosphatases (PAP3, PAP2) were found to be upregulated [15, 42]. Changes in expression were also observed in transcription factor families related to stress responses, such as WRKY, bHLH, MYB, and AP2/ERF [39]. Furthermore, genes belonging to the AUX/IAA family, which are associated with auxin responses, showed significant downregulation (Fig. S2). On the other hand, it is possible that the long-term deficiency of Pi, which is a major component of DNA and RNA, limits the synthesis of internal DNA and RNA in plants, thereby reducing the available transcriptional information. The high proportion of downregulated genes among the DEGs and the upregulation of the ‘Purine metabolism’ pathway, which is a crucial component of genetic information, provide some evidence to support this hypothesis.

#### Transcriptional differences in *E. Pubescens* leaves under phosphorus deficiency stress for 30d and 90d

Based on the criteria of *p*-adj < = 0.05 and |log_2_FC| >= 1.0, differentially expressed genes (DEGs) were identified in the leaves of *E. pubescens* subjected to phosphorus deficiency for 30 days and 90 days. A total of 3209 DEGs were obtained. In comparison to the 30d -P treatment, 988 DEGs were up-regulated, and 2221 were down-regulated in the 90d -P treatment (Fig. S1C). This points towards significant alterations in the transcriptional profile of *E. pubescens* leaves under different durations of phosphorus deficiency stress. Compared to the 30d -P treatment, a majority of genes exhibited down-regulation in the 90d -P treatment, potentially influenced by upstream transcription factors (TFs) or microRNAs.

GO and KEGG enrichment analyses were conducted on the DEGs. The GO enrichment results indicated upregulation in categories such as ‘cell communication’, ‘defense response’, ‘protein serine/threonine kinase activity’, and ‘sequence-specific DNA binding’, while downregulation was observed in categories like ‘cell wall’, ‘cellular carbohydrate metabolic process’, and ‘cell periphery’ in *E. pubescens* leaves. The KEGG enrichment results revealed upregulation in pathways such as ‘Tryptophan metabolism’, ‘Valine, leucine and isoleucine biosynthesis’, and ‘Ribosome biogenesis in eukaryotes’, while downregulation was evident in pathways like ‘Cutin, suberine and wax biosynthesis’ and ‘Amino sugar and nucleotide sugar metabolism’ (Table S6).

**Fig. 7:**
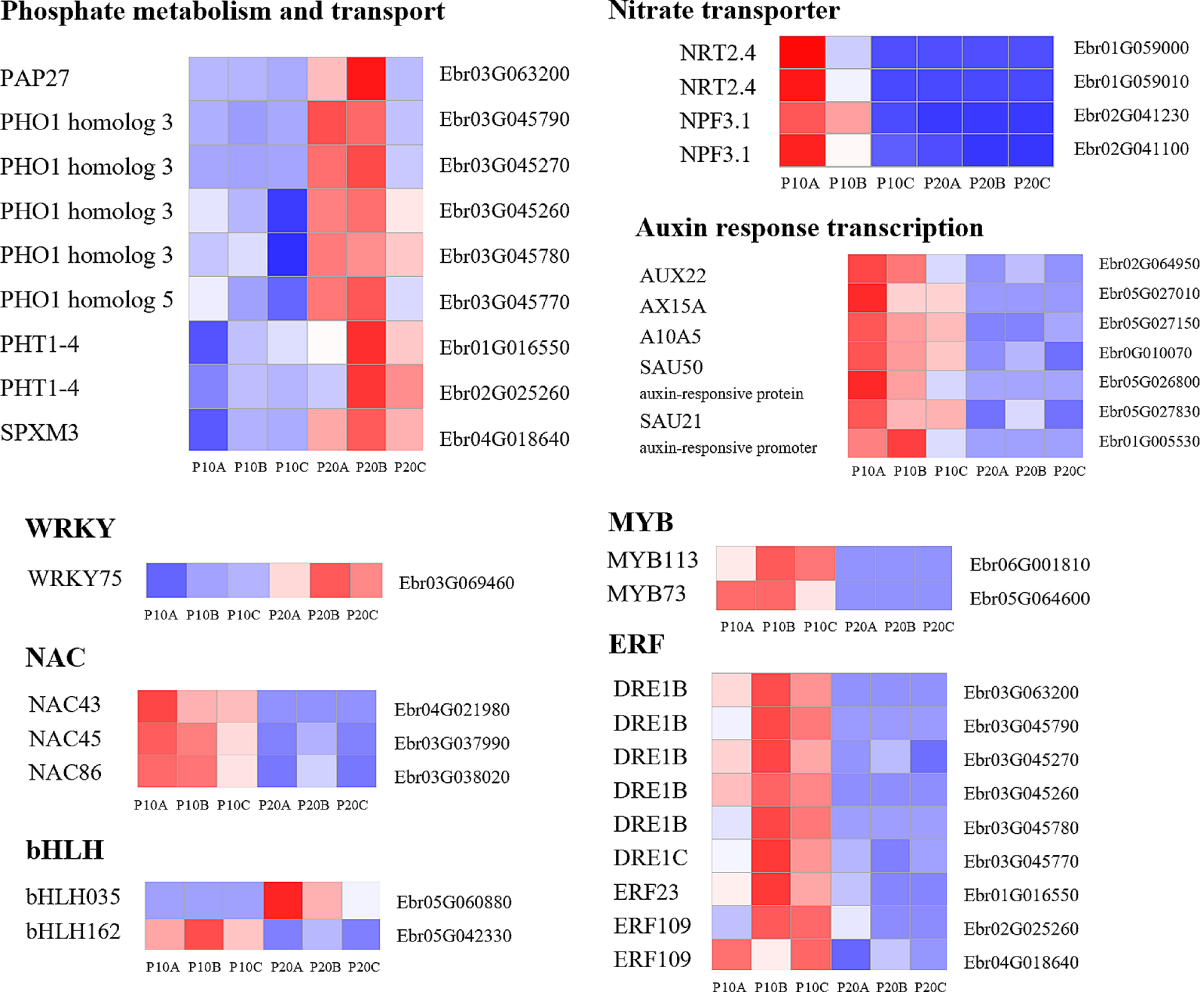
Compared with phosphorus deficiency stress for 30 days, the heatmap of gene expression related to phosphorus metabolism and transport, NRT, auxin response, and TF family genes in *E. pubescens* leaves under phosphorus deficiency stress for 90 days (with |log_2_FC| > 5.0). The gene annotations are on the left side of the heatmap, and the gene IDs are on the right side. P10: 30d -P treatment, P20: 90d -P treatment, and A, B, C are different duplicates

DEGs with |log_2_FC| >= 5.0 demonstrated extremely significant differences in *E. pubescens* leaves subjected to phosphorus deficiency stress for 30 days and 90 days, potentially serving as key genes in *E. pubescens*’ response to prolonged phosphorus stress (90 days). Notably, genes associated with phosphorus metabolism, phosphorus transporters, and phosphatases (PAP27, PHO1 homolog 3, PHO1 homolog 5, PHT1-4, SPXM3) exhibited significant upregulation, while genes linked to nitrate transport (NRT2.4, NPF3.1 [43]) displayed downregulation (Fig. 7). This implies that extended phosphorus deficiency stress impacts nitrogen absorption and assimilation in *E. pubescens* leaves. Additionally, in comparison to the 30d -P treatment, auxin-related response genes witnessed downregulation in the 90d -P treatment, indicating varying degrees of growth limitation at different stages of phosphorus deficiency stress, with an increased growth inhibition under prolonged phosphorus deficiency stress.

Moreover, a total of 171 transcription factors (TFs) spanning 38 TF families were identified among all DEGs. Within the TFs with |log_2_FC| > 5.0, representatives from the WRKY family (WRKY75 was down-regulated), NAC family (NAC45, NAC43, and NAC86 were all down-regulated), bHLH family (bHLH035 was up-regulated and bHLH162 was down-regulated), MYB family (MYB113 and MYB73 were both down-regulated), and ERF family (all were down-regulated) were observed (Fig. 7). These transcription factors play a pivotal role in orchestrating the transition of *E. pubescens* leaf strategies from short-term phosphorus deficiency to prolonged phosphorus deficiency stress, positively or negatively regulating the expression of downstream genes.

### Analysis of miRNAs and target genes in *E. Pubescens* leaves under phosphorus deficiency stress

In this investigation, miRNAs extracted from *E. pubescens* leaves underwent analysis under different phosphorus levels at 30 days and 90 days. A total of 2.3 GB of raw data were collected from 12 samples (including three replicates). Following the exclusion of reads with sequencing adapters or low sequencing quality, further removal of rRNA, tRNA, snRNA, and repeat sequences yielded 18,662,013 clean reads. The lengths of miRNAs in each sample ranged from 18 to 32 nucleotides (nt), with the most prevalent miRNAs being 21 nt in length (Fig. S2).

**Fig. 8:**
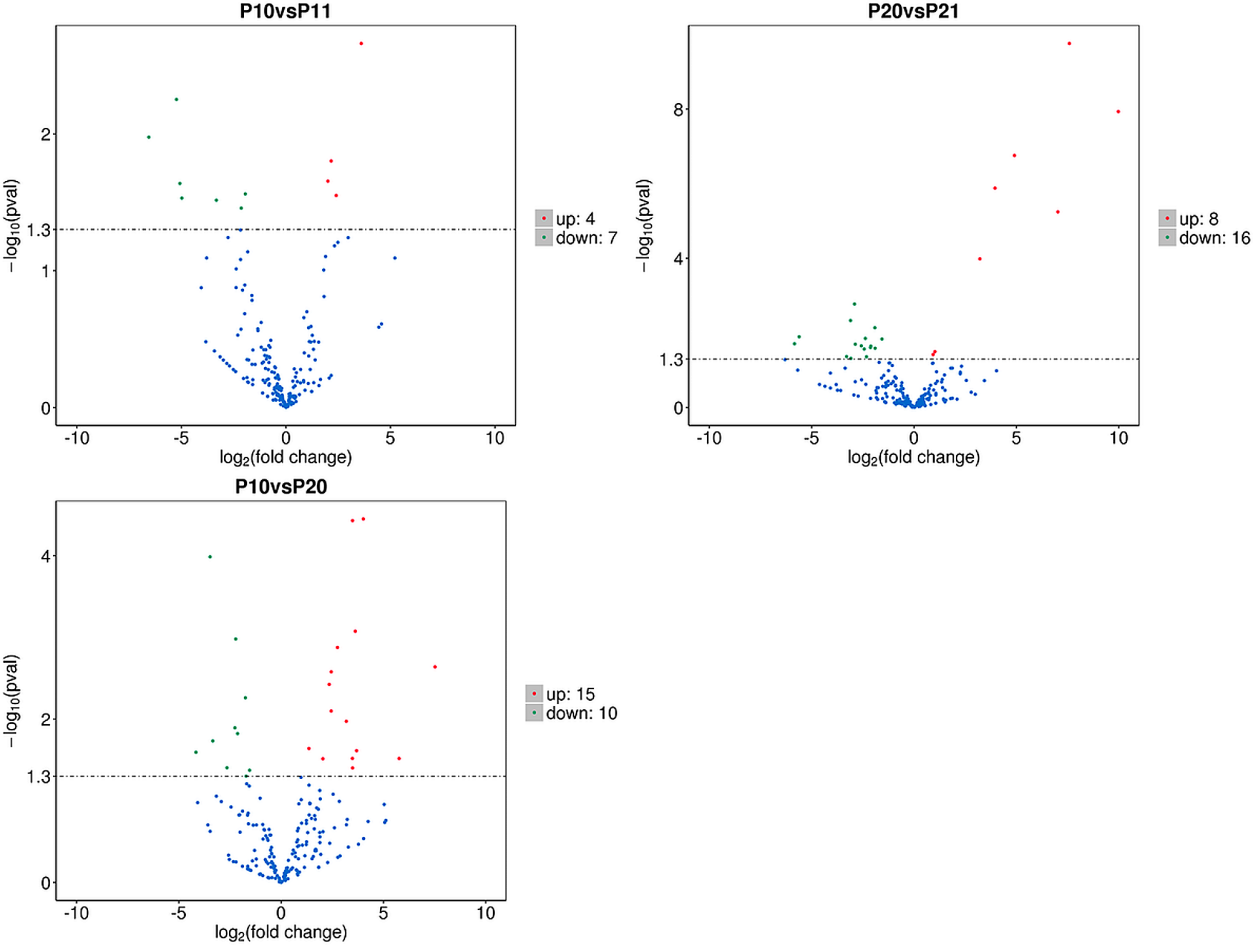
Differential miRNAs under varying phosphorus treatments at the same time point and under the same phosphorus treatment at different time points based on the criterion of *p*-val < 0.05 (Volcano plot, P10: 30d -P treatment, P11: 30d + P treatment, P20: 90d -P treatment, P21: 90d + P treatment)

In comparison to the 30d + P treatment, 11 novel miRNAs were identified as differentially expressed in the 30d -P treatment, with 4 upregulated and 7 downregulated. When contrasting the 90d -P treatment with the 90d + P treatment, 24 differentially expressed miRNAs were detected, including 3 known miRNAs and 21 novel miRNAs. Among these, 8 were upregulated, and 16 were downregulated. Similarly, when comparing the 30d -P treatment to the 90d -P treatment, 25 differentially expressed miRNAs were recognized, encompassing 2 known miRNAs and 23 novel miRNAs. Of these, 15 were upregulated, and 10 were downregulated (Fig. 8).

**Fig. 9:**
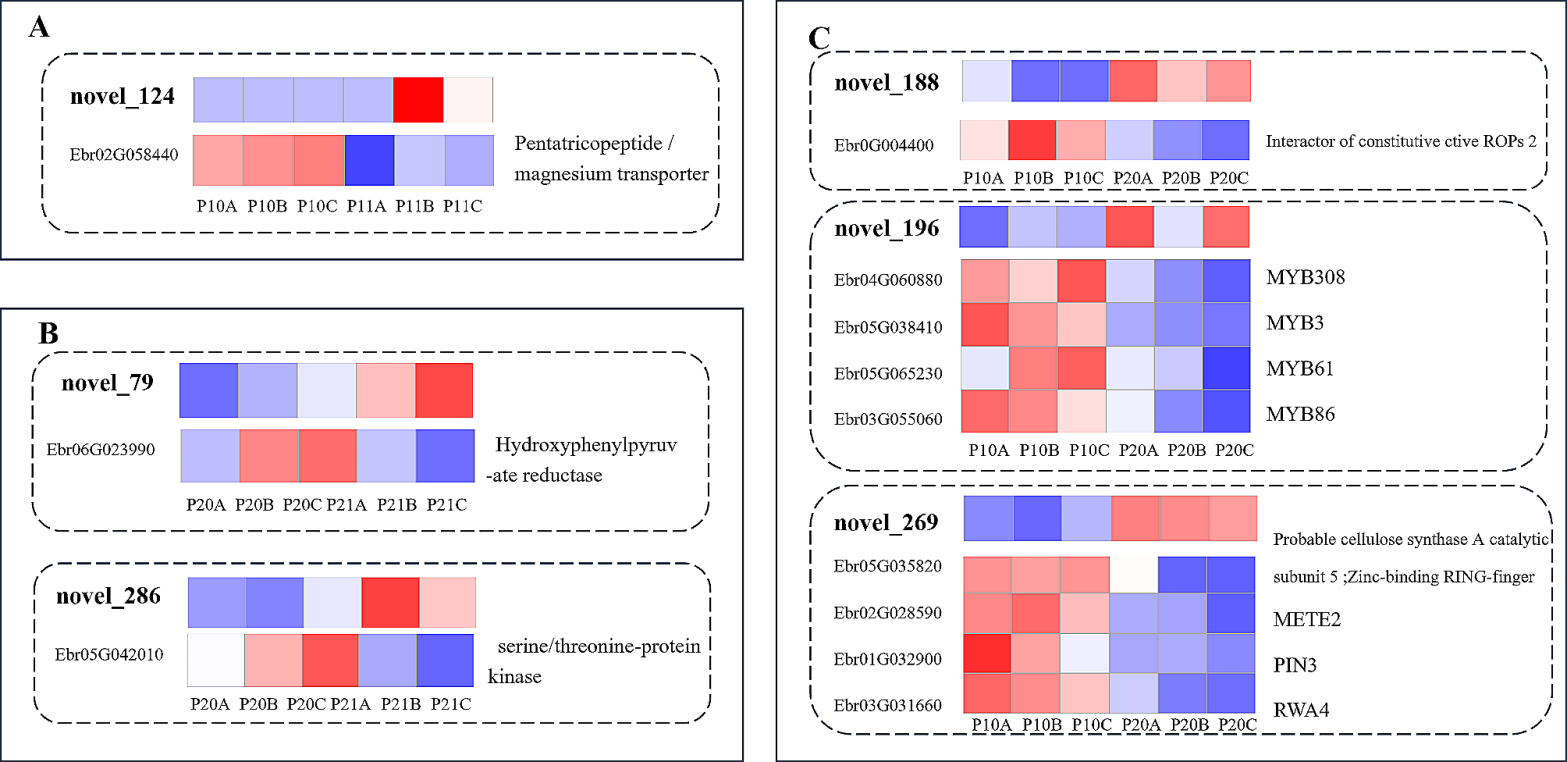
Heatmap of differentially expressed miRNAs and their target genes under different phosphorus treatments at the same time point and different time points under phosphorus deficiency stress. The expression levels of miRNAs are calculated using TPM, and the expression levels of mRNAs are calculated using FPKM. The miRNA IDs are bold, the gene IDs are on the left side of the heatmap, and the gene annotations are on the right side. **A**) P10 (30d -P) vs. P11 (30d + P); **B**: P20 (90d -P) vs. P21 (90d + P); C: P10 (30d -P) vs. P20 (90d -P)

**Fig. 10:**
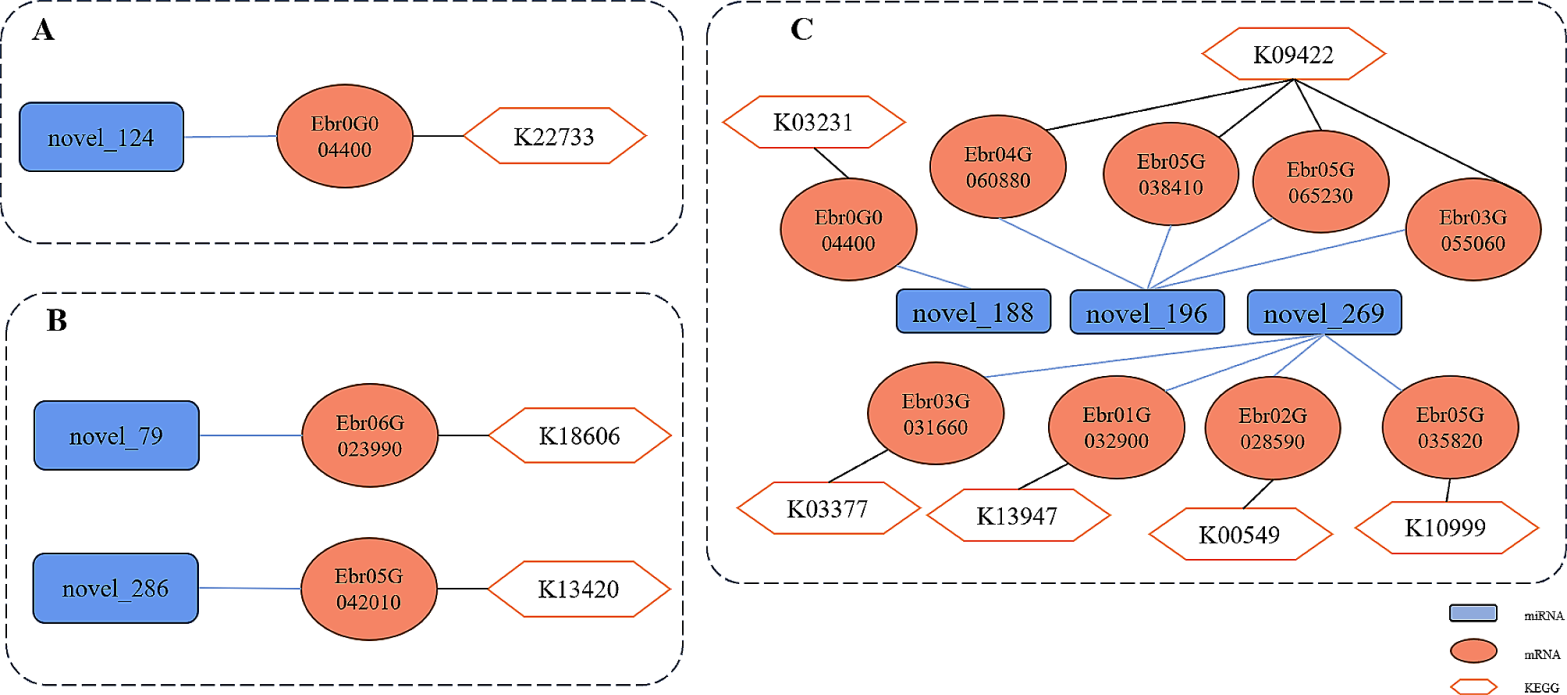
Network diagram of differentially expressed miRNAs and their target genes with KEGG annotations under different phosphorus treatments at the same time point and different time points under phosphorus deficiency stress. **A**: P10 (30d -P) vs. P11 (30d + P); **B**: P20 (90d -P) vs. P21 (90d + P); **C**: P10 (30d -P) vs. P20 (90d -P)

Upon comparing miRNA expression alterations in leaves subjected to 30d -P and 30d + P treatments, one differential miRNA and one target gene were unveiled (Figs. 9A and 10A). Under the 30d -P treatment, novel_124 demonstrated significant downregulation, resulting in the upregulation of its target gene Ebr02G058440, identified as Pentatricopeptide. Pentatricopeptide is implicated in various roles in plant gene expression and physiological processes, overseeing plant growth, development, and defense responses to stress [44].

In the comparison between 90d -P and 90d + P treatments, two differential miRNAs and two target genes were obtained (Figs. 9B and 10B). Novel_79 was significantly downregulated, resulting in the upregulation of its target gene Ebr06G023990, identified as Glyoxylate/hydroxypyruvate/pyruvate reductase 2KGR, involved in tyrosine metabolism [45]. Additionally, novel_286 was significantly downregulated, leading to the upregulation of its target gene Ebr05G042010, identified as MDIS1-interacting receptor-like kinase 2, which activates MAPK signaling pathways and various defense responses against stress [46]. Furthermore, miR399 was identified as significantly upregulated under the 90d -P treatment, but its differentially expressed target gene Ebr03G015380 encoded an uncharacterized protein.

When comparing the 30d -P and 90d -P treatments, three differential miRNAs and their corresponding nine target genes were identified (Figs. 9C and 10C). Under the 90d -P treatment, novel_188, novel_196, and novel_269 were upregulated, leading to the downregulation of their respective target genes. Specifically, the target gene of novel_188, Ebr0G004400, was identified as Interactor of constitutive active ROPs 2. ROPs 2 plays a crucial role as a molecular switch in signal transduction processes involved in plant morphogenesis, hormone regulation, stress responses, and other vital plant activities [47]. The downregulation of this target gene may be related to signal shifts in plant stress responses. The target genes of novel_196: Ebr04G060880, Ebr05G038410, Ebr05G065230, and Ebr03G055060, were identified as MYB308, MYB3, MYB61, and MYB86, respectively, belonging to the MYB family, a common plant transcription factor family responding to stress. Novel_269 had four valid target genes, including Ebr05G035820, Ebr02G028590 encoding cellulose synthase A catalytic subunit 5 and secondary wall acyltransferase, respectively, affecting cell wall formation and thickening. The target gene Ebr01G032900 encodes METE2 involved in methionine synthesis, while Ebr03G031660 encodes the auxin efflux protein PIN3, affecting plant growth and development in response to auxin [48].

### Validation of differentially expressed genes through RT-qPCR

**Fig. 11:**
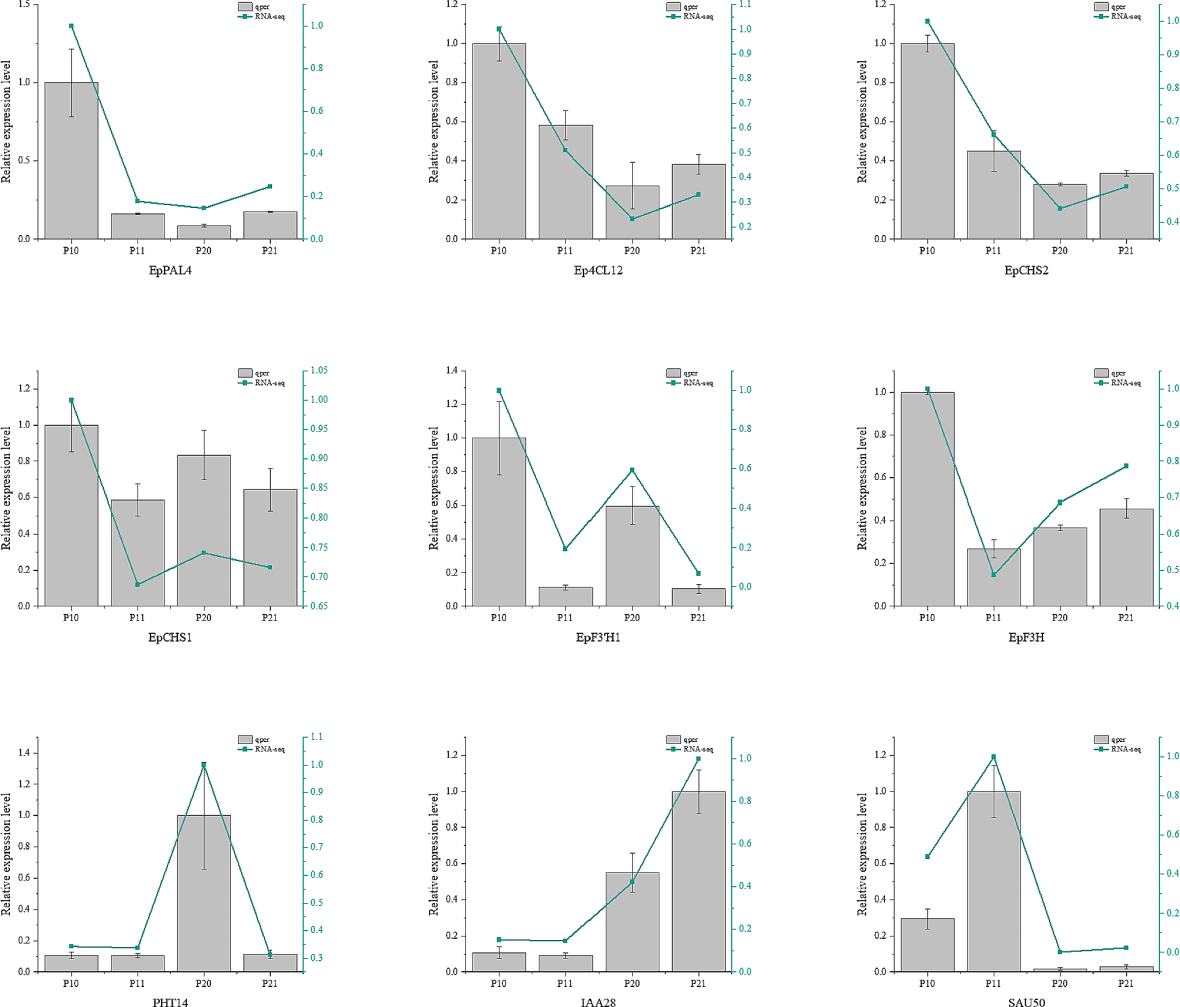
mRNA expression levels were verified by RT-qPCR, P10: 30d -P treatment, P11: 30d + P treatment, P20: 90d -P treatment, P21: 90d + P treatment

**Fig. 12:**
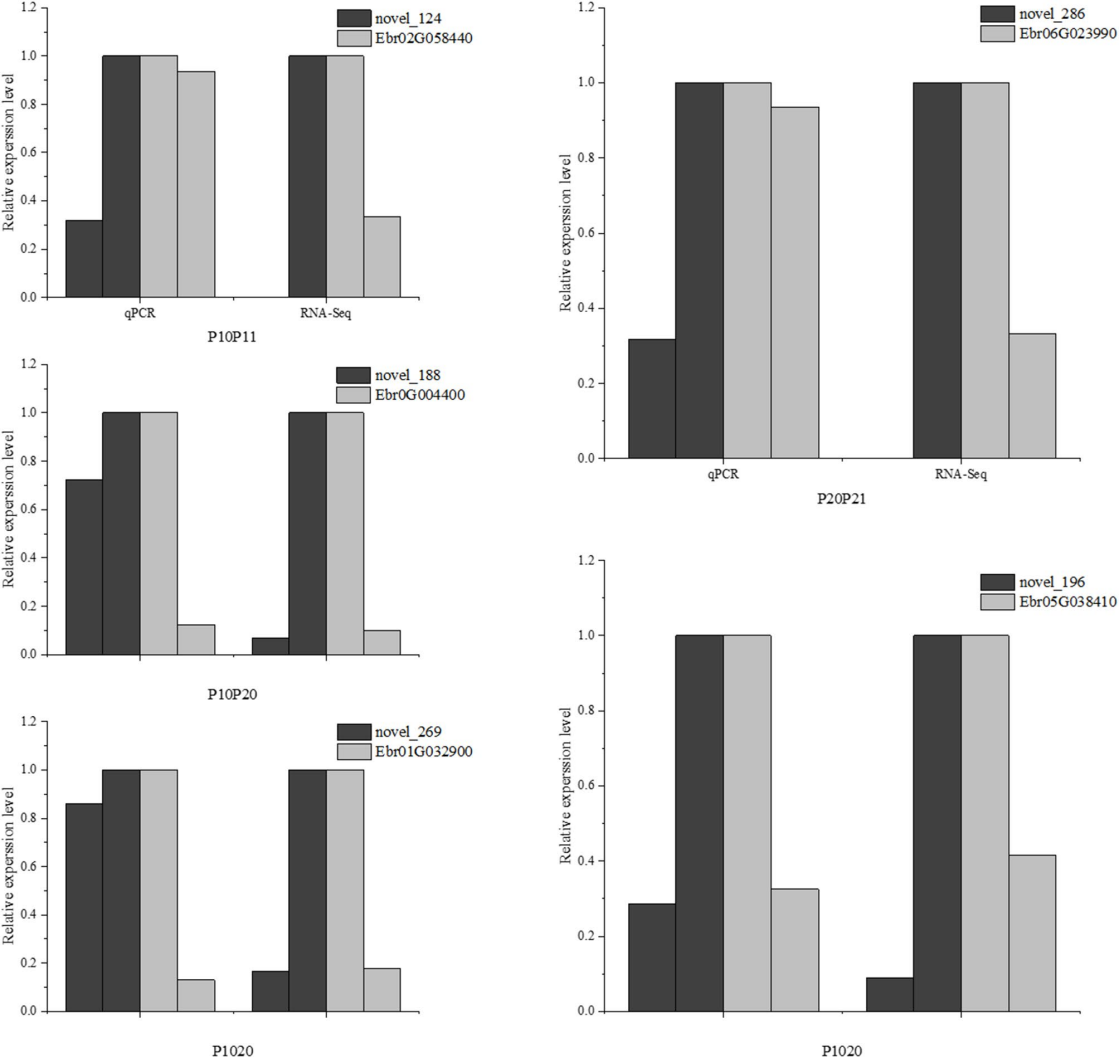
Expression levels of miRNA and its target mRNA were verified by RT-qPCR

In order to validate the accuracy of the RNA-Seq findings, quantitative real-time polymerase chain reaction (RT-qPCR) was conducted to assess the transcript levels of identified differentially expressed genes across various treatments. Specifically, pivotal genes associated with flavonoid biosynthesis, the phosphorus transporter PHT1-4, and auxin response-related genes were chosen for validation. The RT-qPCR results unequivocally demonstrated that the expression patterns of the selected 9 differentially regulated genes were in concordance with the transcriptomic data, thereby substantiating the reliability of the RNA-Seq results employed in this investigation (Fig. 11). The miRNA validation results are presented in Fig. 12. The qRT-PCR results showed that the expression changes of the miRNA and its target gene were opposite, and consistent with the sequencing results.

## Discussion

The significance of phosphorus as a vital nutrient in promoting plant growth, development, and biomass accumulation has been extensively studied. Phosphorus deficiency is a significant factor hindering the growth of various plants, such as corn [49] and rice [50]. However, regions where *E. pubescens* is prevalent often face challenges related to phosphorus deficiencies,, including low soil phosphorus levels and inadequate forms phosphate for plant absorption. These obstacles can impede the growth and quality of *E. pubescens*. In order to adapt to this unfavorable environment, *E. pubescens* has progressively evolved an array of strategies to contend with phosphorus deficiency stress. The current work investigated the phenotypic and physiological alterations in *E. pubescens* over different time scales in reaction to phosphorus deficiency. Furthermore, it integrated transcriptomic and miRNA analyses to further investigate how *E. pubescen*s responds to phosphorus deficiency stress.

Plant growth, development, and biomass accumulation were found to be significantly inhibited under conditions of phosphorus deficiency in this study. This inhibition was visually characterized by a reduction in leaf number, diminished leaf area, and an increase in root length, volume, and surface area. That plants their growth strategies in phosphorus-deficient settings These findings imply that plants might modify their growth strategies in phosphorus-deficient environments in order to reduce nutrient demand and increase the surface area available for nutrient absorption (Fig. 1A, B). Since roots are a plant’s primary source of phosphorus, their growth helps to increase the efficiency with which phosphorus is absorbed, thereby reducing the stress that results from phosphorus shortage in plants [44]. Moreover, the insufficiency of phosphorus stress inhibited the photosynthetic activity of *E. pubescens*, leading to a notable decline in chlorophyll levels and total biomass, with a concurrent rise in the root biomass proportion (Fig. 1C and D). This could potentially be an adaptive survival strategy used by plants in response to nutrient-limited conditions. The roots of plants are essential for the uptake of nutrients, and an increased root biomass can be advantageous in aiding plants in locating additional nutrients and adapting to challenging environmental conditions [51, 52].

Photosynthesis, serving as the primary phase of plant carbon metabolism, provides carbon sources and energy for various metabolic processes and biomass accumulation throughout the plant’s life cycle [53]. Prior studies have demonstrated that a lack of phosphorus can impede photosynthesis in plants and impact the accumulation of carbohydrates, typically due to disrupted electron transport or impairment of associated photosynthetic sites [4, 14, 50, 54]. The findings from the transcriptome analysis elucidate this alteration at the molecular level. In comparison to the application of + P, the -P treatment resulted in a decrease in the expression of the ‘photosystem II oxygen evolving complex’ GO category after 30 days (Table S3). The oxygen-evolving complex (OEC) is pivotal in the process of photosynthesis, facilitating the light-driven oxidation of water to produce molecular oxygen and protons, as well as electron transportation [55]. The reduction in the expression of genes associated with this complex hinders the flow of electrons in photosynthesis. Furthermore, the levels of MTEF4 and MTEF6, which are crucial transcription termination factors essential for the development of 16 S rRNA and 23 S rRNA in chloroplasts [56], were also notably decreased, impacting the maturation of chloroplasts, the primary location for photosynthesis, consequently leading to a decrease in photosynthetic efficiency and carbohydrate production (Fig. 1D, Table S3).

Despite the decrease in photosynthesis in plants facing phosphorus deficiency stress, research indicates that there may be an enhancement in carbohydrate metabolism. This phenomenon has been observed in various plant studies focusing on phosphorus deficiency [57]. For instance, cotton plants exhibit reduced photosynthesis under phosphorus stress, yet show increased activities of enzymes and gene expression related to carbohydrate metabolism [16]. The impact of phosphorus deficiency stress on carbohydrate metabolism in E. pubescens appears to align with the response strategy observed in cotton under similar conditions. This impact is not only evident in the photosynthetic capacity but also in the leaves’ ability to mobilize stored carbohydrates when photosynthesis is insufficiently effective [16]. In the current investigation, compared to the treatment with sufficient phosphorus, GO categories associated with carbohydrate metabolism, such as ‘polysaccharide metabolic process’, ‘cellular glucan metabolic process’, and ‘carbohydrate catabolic process’, were found to be upregulated in the 30d -P treatment (Table S3). Similarly, the ‘starch and sucrose metabolism’ pathway was significantly upregulated in the 90d -P treatment (Table S5). These carbohydrates involved may have accumulated in the plant prior to stress and could also accumulate due to reduced carbohydrate utilization resulting from a more pronounced inhibition of plant growth compared to photosynthesis [58]. Furthermore, upregulation of carbohydrate metabolism may help the plant respond to phosphorus starvation by providing energy or acting as a signaling mechanism to regulate the plant’s response to phosphorus deficiency stress [16, 57].

Moreover, plant hormones serve as critical signaling molecules that regulate plant growth, development, and overall morphological structure [59, 60]. They also play a pivotal role in plant responses to environmental stress [59, 60]. Phosphorus deficiency stress has been shown to notably decrease the tiller count in plants [5], which is regulated by the strong interaction between internal phosphorus content and hormones such as strigolactones [61], auxin [62], and cytokinin. In investigations on phosphorus deficiency stress in peanuts, a substantial decrease in the content of IAA, ETH, and CTK in roots was observed, accompanied by an increase in ABA content, with an opposite trend noted in leaves. This distinct hormonal regulation in different plant parts can lead to modifications in GS and NR activity in roots and leaves, influencing the balance of nitrogen metabolism and plant growth morphology [14]. Findings from the study demonstrate a significant decline in phosphorus content and accumulation in leaves under -P treatment (Fig. 1E). The results of our study indicate that under -P treatment, the phosphorus content and accumulation in leaves decrease significantly (Fig. 1E). Compared with + P tremeat, the ‘Plant hormone signal transduction’ pathway is notably upregulated during the 30d -P treatment (Table S3), while the ‘Steroid biosynthesis’ pathway is markedly downregulated in the 90d -P treatment (Table S5). This suggests that phosphorus deficiency stress exerts complex effects on the growth and development of *E. pubescens* by influencing hormonal signaling changes. Compared to the 30d -P treatment, genes related to auxin response and transcription factors are significantly downregulated under 90d -P treatment (Fig. 7), indicating that with prolonged phosphorus deficiency phosphorus deficiency stress, the growth inhibition of *E. pubescens* also intensifies.

Biotic or abiotic stress can induce the excessive accumulation of reactive oxygen species (ROS) in plants, resulting in oxidative damage to the plasma membrane system [63, 64]. Flavonoids, which are the primary bioactive components of Epimedium, possess the ability to scavenge ROS in plants, thereby improving resistance to various stresses such as nutrient deficiency and high light intensity [65–67]. A study conducted on soybeans subjected to phosphorus starvation revealed an increase in the levels of 26 flavonoid-related metabolites [68]. Similarly, *Artemisia argyi* leaves exposed to low phosphorus stress showed a notable rise in phenolic and flavonoid compounds, along with an upregulation of gene expression encoding crucial enzymes in their metabolic pathways [13]. Moreover, in tobacco leaves experiencing phosphorus deficiency, the transcription levels of genes responsible for flavanol biosynthesis were significantly upregulated [69]. The results obtained from ultra-performance liquid chromatography (UPLC) detection(Fig. 2A and B) in this study revealed that phosphorus deficiency stress significantly promoted the accumulation of Icariin-flavonoids in leaves. Concurrently, transcriptional results indicated a significant upregulation of genes related to the ‘Flavonoid biosynthesis’ pathway under the 30d -P treatment (Table S3). Subsequent Gene Set Enrichment Analysis (GSEA) of this pathway of this pathway (Fig. 3), along with previous research findings, facilitated the construction of a graphical representation (Fig. 4) depicting the alterations in expression levels of key genes involved in the biosynthesis of Icariin-flavonoids in *E. pubescens* leaves under phosphorus deficiency stress.

Moreover, it was observed that various gene families including WRKY, NAC, ERF, bHLH, and MYB were implicated in regulating the expression of downstream genes in *E. pubescens* leaves under different phosphorus deficiency stress stages. Among them, the bHLH family, often serving as an auxiliary factor, works synergistically with the MYB family to cope with various stresses and regulate flavonoid synthesis [70]. MYB60 is responsive to drought stress and changes in ABA hormone levels, and it negatively regulates the flavonoid pathway [71]. Compared to the + P treatment, the expression of MYB60 and the bHLH family was downregulated under the 30d -P treatment (Fig. 5), leading to an upregulation of the expression of genes related to flavonoid synthesis under phosphorus deficiency stress. Additionally, investigations on rapeseed, corn, and soybeans under phosphorus deficiency stress demonstrated that plants can mitigate the accumulation of ROS through glutathione metabolism [12, 72, 73]. Similar findings were observed in this study, where the ‘Glutathione metabolism’ pathway was upregulated during the later stages of phosphorus deficiency stress (90d -P treatment).

PHT, PHO, SPX domain proteins, and purple phosphatase represent proteins and enzymes pivotal in phosphorus metabolism and transport in plants. When subjected to severe phosphorus deficiency stress, Switchgrass demonstrates a significant increase in the expression of genes responsible for encoding PHT, PHO, and SPX domain proteins [74]. Similarly, in soybean leaves, the expression of purple acid phosphatase is notably up-regulated under conditions of phosphorus deficiency [12, 15]. The induction of these proteins and enzymes under phosphorus deficiency conditions aims to enhance phosphorus absorption and transport. Studies have shown that lipid remodeling is a common internal phosphorus recycling pathway in plants. To alleviate phosphorus deficiency stress, plants synthesize glycolipids and sulfolipids to replace some phospholipids on membranes, thereby releasing phosphorus. Studies have elucidated lipid remodeling as a prevalent phosphorus recycling pathway, where glycolipids and sulfolipids are synthesized to replace some phospholipids on biological membranes, releasing phosphorus to alleviate phosphorus deficiency stress. This research demonstrates that 90d -P treatment leads to the increased expression of genes encoding PHT1-4, PHO1 homolog3, SPX domain proteins, purple acid phosphatase (Fig. 7), and the ‘sulfate transmembrane transporter activity’ GO category (Table S7) in *E. pubescens* leaves, as compared to the 30d -P treatment. This upregulation enhances phosphorus metabolism, transport, and internal phosphorus cycling [75, 76]. Moreover, the ‘Tryptophan metabolism’ pathway is up-regulated (Table S7), promoting the glycosylation of membrane lipids, replacing phospholipids, and releasing phosphorus. Previous studies have indicated a substantial phosphorus content in the pectin of the cell wall. In response to phosphorus deficiency stress, plants utilize enzymes like polygalacturonase to decompose the cell wall, enabling phosphorus reuse and increasing the available phosphorus content [77, 78]. Notably, this study observed an upregulation of the ‘polygalacturonase activity’ GO category (Table S5) under the 90d -P treatment compared to the + P treatment. Furthermore, the ‘cell wall macromolecule catabolic process’ pathway was upregulated in the 90d -P treatment relative to the 30d -P treatment, while the ‘cell wall’ and ‘cell periphery’ GO categories were downregulated (Table S7). These findings suggest that *E. pubescens* breaks down the cell walls in its leaves during the later stages of phosphorus deficiency stress using enzymes like polygalacturonase to redistribute phosphorus resources and sustain essential physiological functions [77, 78]. Furthermore, miRNA results indicate that novel_269 inhibits cell wall formation and thickening by down-regulating the expression of target genes Ebr05G035820 and Ebr02G028590, which encode cellulose enzyme A catalytic subunit 5 and secondary wall acyltransferase, respectively (Fig. 10). In summary, as the duration of phosphorus deficiency treatment extends to 90 days, plants may gradually adapt their phosphorus deficiency response mechanisms by adjusting transcriptional and post-transcriptional regulatory strategies, thereby more effectively coping with long-term phosphorus deficiency stress.

These findings indicate that the transcriptional response mechanism in *E. pubescens* leaves mirrors that of most plants, encompassing (1) hormone signal transduction, (2) photosynthesis and carbohydrate metabolism, (3) secondary metabolism (such as flavonoid synthesis), and (4) alterations in transcripts related to phosphorus metabolism, transport, and recycling. Similar to observations in Switchgrass, this suggests that the transcriptional responses of plants under phosphorus deficiency stress are generally conserved, emphasizing the pivotal role of transcriptional regulation in plant responses to phosphorus deficiency stress [74]. Additionally, miRNA sequencing results revealed that, compared to the 30d -P treatment, a greater number of miRNAs were significantly upregulated under the 90d -P treatment. This suggests that miRNA regulation assumes a more prominent role in shaping the transition of the leaf response strategy from short-term to long-term phosphorus deficiency stress.

Furthermore, the comparison between the -P and + P treatments at 30 days and 90 days revealed a notable decrease in the number of differentially expressed genes (DEGs) between the two treatments at 90 days, accompanied by a marked increase in the quantity of downregulated DEGs (Fig. S1B). This phenomenon can be ascribed to the depletion of DNA and mRNA bases caused by phosphorus (Pi) deficiency, along with the enhanced degradation of cell walls and chloroplasts (Table S5). It can be deduced that *E. pubescens* appears to be decomposing its leaves and reallocating resources to sustain other organs for survival. This behavior may represent a distinctive trait of *E. pubescens*, a perennial herb, in reaction to the stress induced by nutrient deficiency. Following leaf shedding, *E. pubescens* can regenerate new leaves in the subsequent year or once the stress subsides. Consequently, it is conceivable that the phosphorus-deficient condition at 90 days signifies a relatively prolonged and stable phase in the advanced stages of phosphorus deficiency stress for *E. pubescens*, which persists until all its leaves are shed or the stress is mitigated. Furthermore, in contrast to the 30d -P treatment, genes associated with auxin responses and transcription factors were notably downregulated in the 90d -P treatment (Fig. 7), whereas no significant downregulation was observed in the comparison between the 30d -P treatment and the + P treatment (Table S3). These findings suggest that *E. pubescens* employs distinct response mechanisms under short-term (30 days) and long-term (90 days) phosphorus deficiency stress. While the strategy during short-term stress focuses on growth maintenance, the approach shifts towards survival preservation during long-term stress.

## Conclusion

**Fig. 13:**
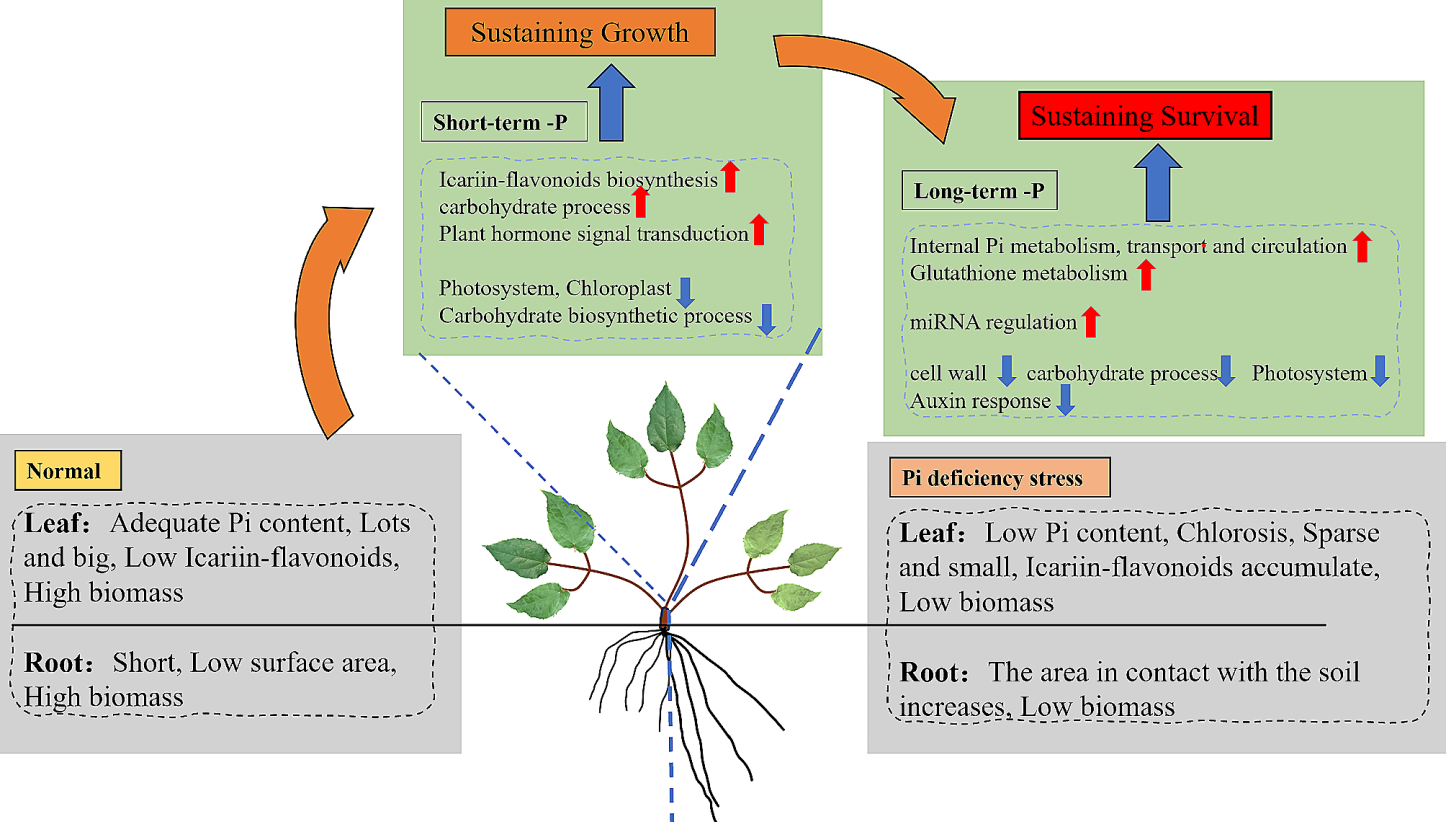
E. pubescens response mechanism to phosphorus deficiency stress

*E. pubescens* exhibits a dynamic and multifaceted response to phosphorus deficiency, involving adjustments in growth morphology, alterations in physiological metabolism, and molecular changes. The impact of phosphorus deficiency stress is evident in the promotion of root growth, contrasting with a significant inhibition of leaf (the medicinal part of *E. pubescens*) growth. Despite this inhibition, there has been an increase in the accumulation of active ingredients. Furthermore, there are discernible changes in intrinsic transcription and miRNA expression in response to phosphorus deficiency in the leaves. In the early stage of phosphorus deficiency stress (30 days), *E. pubescens* leaves demonstrate adaptive responses by generating sufficient energy, scavenging reactive oxygen species (ROS), and adjusting plant morphology. This is achieved through the upregulation of genes related to carbon metabolism, flavonoid synthesis, and hormone signal transduction pathways. These adjustments serve as coping mechanisms for short-term phosphorus deficiency and sustain its growth. However, as the stress duration extends to 90 days, entering the later stage of phosphorus deficiency stress, plant growth is further inhibited and the plant adopts more complex adaptive strategies to promote phosphorus cycling and recycling in the leaves (upregulating the expression of PHT1-4, PHO homolog3, etc.), enhance transcriptional changes and post-transcriptional regulation (miRNA regulation and protein modification), and gradually begin to discard and decompose leaves to resist the challenges of long-term phosphorus deficiency stress and sustain survival. The comprehensive transcriptional analysis results presented in Fig. 13 offer valuable insights into understanding the intricate response mechanisms of *E. pubescens* leaves under phosphorus deficiency stress. Furthermore, they provide potential target genes for breeding *E. pubescens* genotypes that are tolerant to low phosphorus, contributing to advancements in plant breeding strategies.

### Electronic supplementary material

Below is the link to the electronic supplementary material.

**Supplementary Material 1: Table S1**. Nutrient solution formulations for treatment with different phosphorus levels. **Table S2**. qPCR genes and primer information. **Table S3**. P10vsP11 Upward and down-regulated gene GO and KEGG enrichment results. **Table S4**. P10 vs. P11 FLAVONOID_BIOSYNTHESIS(ATH00941) GSEA enrichment results. **Table S5**. The GO and KEGG enrichment results of up-regulated and down-regulated genes in P20 vs. P21. **Table S6**. P20vsP21 (*p*-val < 0.05) Upward and down-regulated gene GO and KEGG enrichment results. **Table S7**. P20vsP10 Upward and down-regulated gene GO and KEGG enrichment results

**Supplementary Material 2: Fig. S1**. Differential genes based on *p*-adj < 0.05, |log_2_FC|≥1 at the same time and at different time-P treatments (volcano), A: P10 (30d –P) vs. P11 (30d + P) DEGs, B: P20 (90d –P) vs. P21 (90d + P) DEGs, C: P10 (30d –P) vs. P20 (90d -P) DEGs. **Fig. S2**. Heatmap of differentially expressed genes (DEGs) in E. pubescens leaves under 90-day phosphorus-deficient stress compared to normal phosphorus conditions (p-val < 0.05), along with heatmaps of phosphorus transport and metabolism-related DEGs and major transcription factor (TF) families (gene annotations on the left side of the heatmaps, gene IDs on the right side, P20: 90d-P, P21: 90d + P, A, B, C represent different duplicates). The log2FC is represented by colors. **Fig. S3**. miRNA length distribution in different treatments, P10: 30d -P, P11: 30d + P, P20: 90d -P, P21: 90d + P, A, B, C are different duplicate

## Data Availability

Transcriptome and miRNA data have been successfully uploaded and released to the GEO database for public access and use. The data includes raw sequencing files, which have been verified to ensure their integrity. Other researchers can access the transcriptome sequencing data at https://www.ncbi.nlm.nih.gov/geo/query/acc.cgi?acc=GSE259442 the miRNA sequencing data at https://www.ncbi.nlm.nih.gov/geo/query/acc.cgi?acc=GSE259444.

## Abbreviations

CRD: Completely randomized design
DEGs: Differentially expressed genes
*E. pubescens*: *Epimedium pubescens*
GB: Gigabytes
GO: Gene Ontology
GSEA: Gene Set Enrichment Analysis
KEGG: Kyoto Encyclopedia of Genes and Genomes
miRNA: MicroRNA
OEC: Oxygen-evolving complex
P: Phosphorus
PAP: Purple acid phosphatase
PFGs: Prenylated flavonol glycosides
PHO2: Phosphate starvation response gene PHO2
Pi: Phosphates
+P: Normal phosphorus
-P: Phosphorus deficiency
qRT-PCR: Quantitative Real-time Polymerase Chain Reaction
ROPs 2: Rho-related protein 2
ROS: Reactive Oxygen Species
TF: Transcription factor
UPLC: Ultra-high-performance liquid chromatography
