# Supplementary material for: Exploring the dynamic adaptive responses of Epimedium pubescens to phosphorus deficiency by Integrated transcriptome and miRNA analysis
Source: BMC Plant Biol. 2024 May 30;24:480. doi: 10.1186/s12870-024-05063-y (PMC11138043; doi:10.1186/s12870-024-05063-y)
Supplement: Supplementary file 2 — Supplementary Material 2: Fig. S1. Differential genes based on p-adj < 0.05, |log2FC|≥1 at the same time and at different time-P treatments (volcano), A: P10 (30d –P) vs. P11 (30d + P) DEGs, B: P20 (90d –P) vs. P21 (90d + P) DEGs, C: P10 (30d –P) vs. P20 (90d -P) DEGs. Fig. S2. Heatmap of differentially expressed genes (DEGs) in E. pubescens leaves under 90-day phosphorus-deficient stress compared to normal phosphorus conditions (p-val < 0.05), along with heatmaps of phosphorus transport and metabolism-related DEGs and major transcription factor (TF) families (gene annotations on the left side of the heatmaps, gene IDs on the right side, P20: 90d-P, P21: 90d + P, A, B, C represent different duplicates). The log2FC is represented by colors. Fig. S3. miRNA length distribution in different treatments, P10: 30d -P, P11: 30d + P, P20: 90d -P, P21: 90d + P, A, B, C are different duplicate [file 12870_2024_5063_MOESM2_ESM.docx]

## Supplement


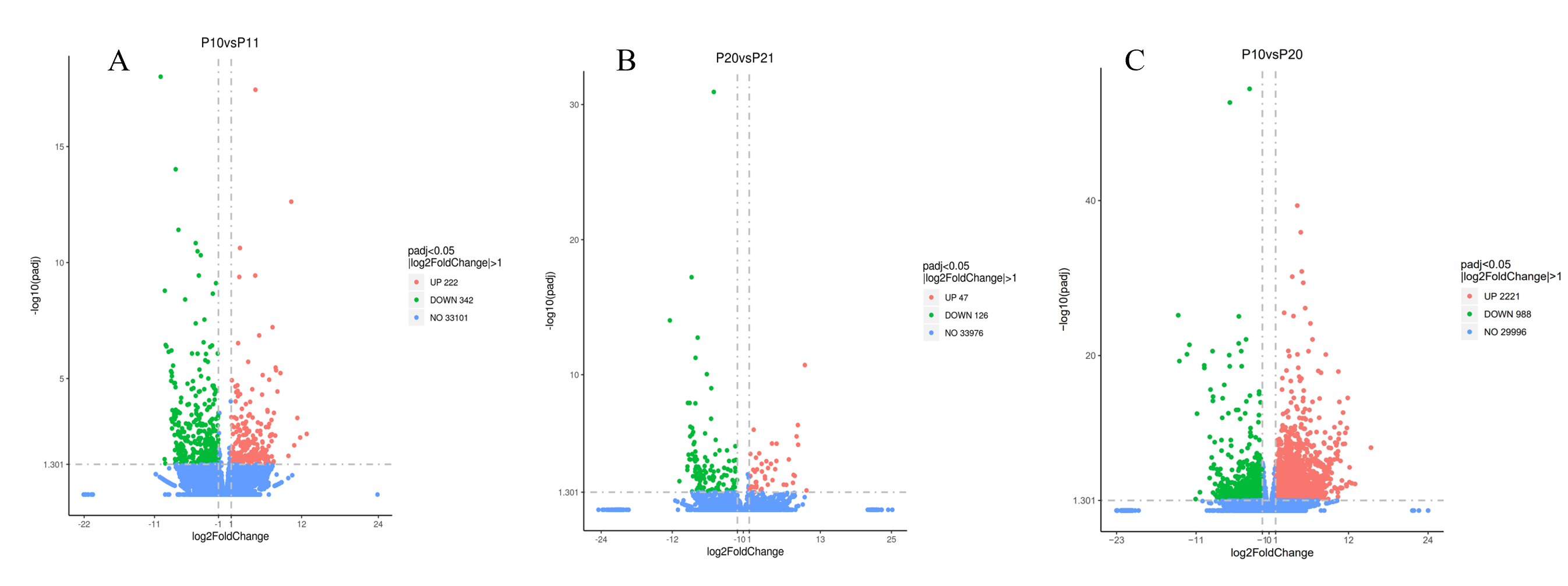


Fig. S1 Differential genes based on *p*-adj <0.05, |log_2_FC|≥1 at the same time and at different time-P treatments (volcano), A: P10 (30d –P) vs P11 (30d +P) DEGs, B: P20 (90d –P) vs P21 (90d +P) DEGs, C: P10 (30d –P) vs P20 (90d -P) DEGs.


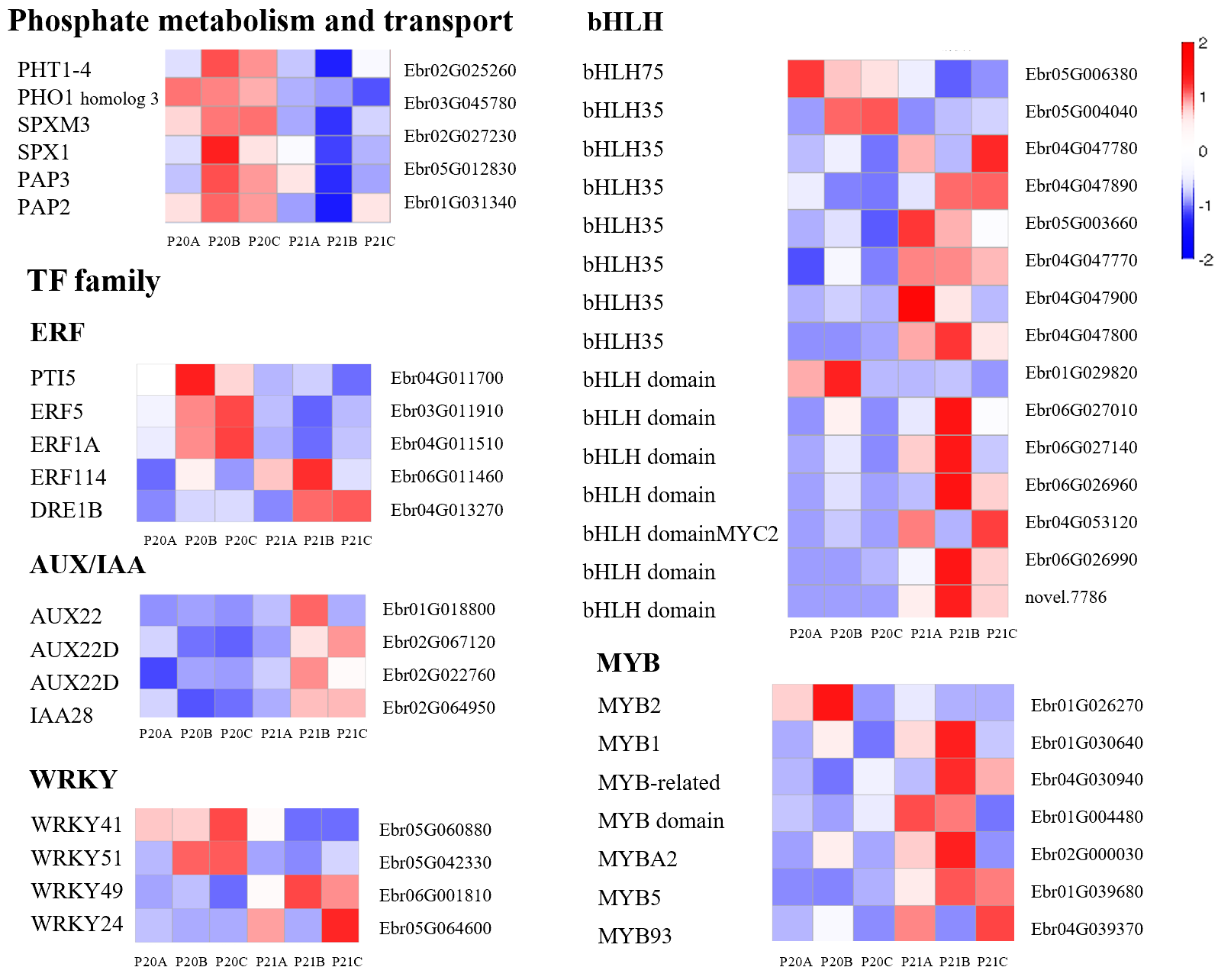


Fig. S2 Heatmap of differentially expressed genes (DEGs) in E. pubescens leaves under 90-day phosphorus-deficient stress compared to normal phosphorus conditions (p-val < 0.05), along with heatmaps of phosphorus transport and metabolism-related DEGs and major transcription factor (TF) families (gene annotations on the left side of the heatmaps, gene IDs on the right side, P20: 90d-P, P21: 90d+P, A, B, C represent different duplicates). The log2FC is represented by colors.


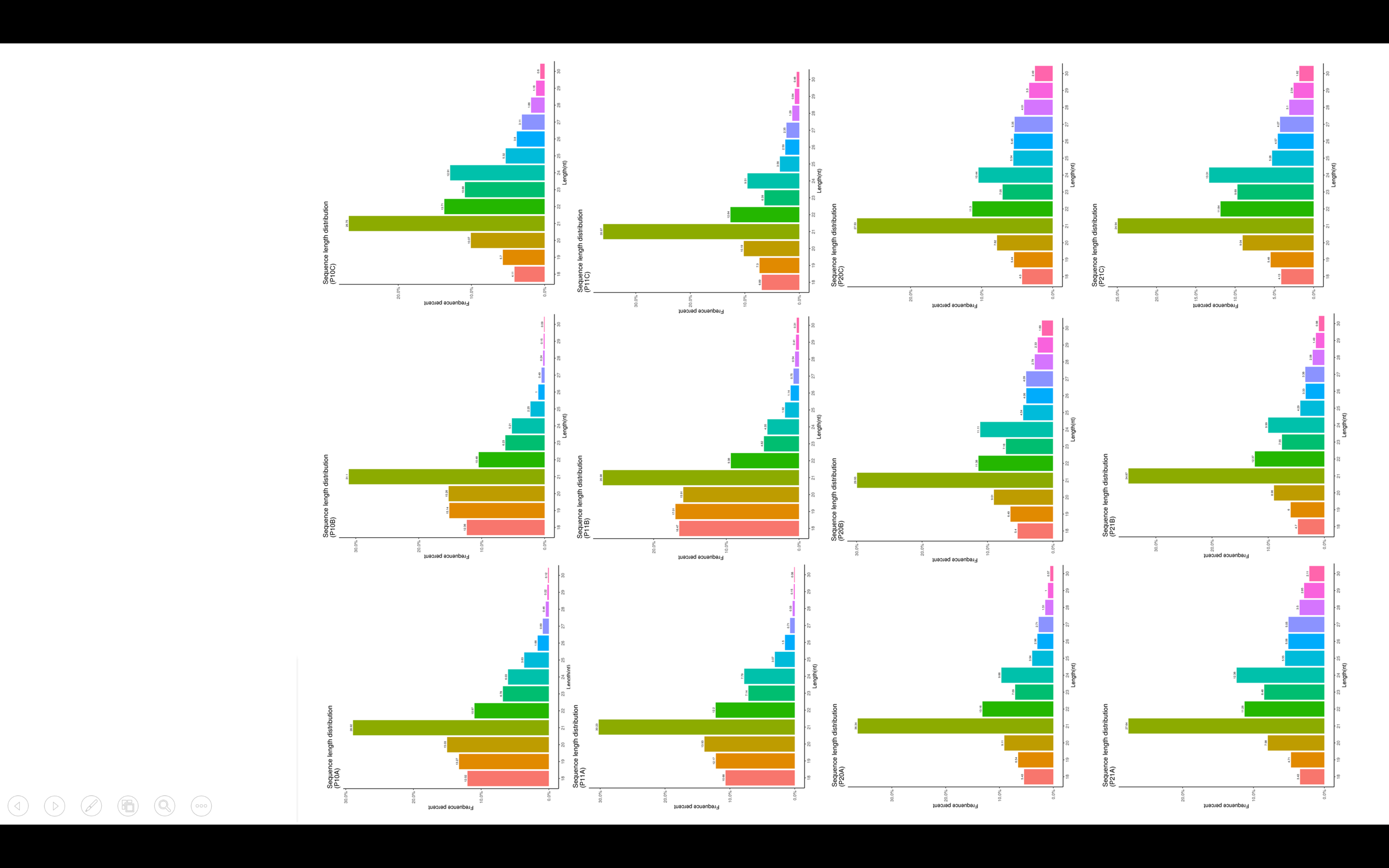


Fig. S3 miRNA length distribution in different treatments, P10: 30d-P, P11: 30d+P, P20: 90d-P, P21: 90d+P, A, B, C are different duplicate.
